# Bacteriobiota and Chemical Changes during the Ripening of Traditional Fermented “Pirot ‘Ironed’ Sausage”

**DOI:** 10.3390/foods12030664

**Published:** 2023-02-03

**Authors:** Svetlana Bogdanović, Slaviša Stanković, Tanja Berić, Igor Tomasevic, Volker Heinz, Nino Terjung, Ivica Dimkić

**Affiliations:** 1 Agriculture and Food College of Applied Studies, Ćirila i Metodija 1, 18400 Prokuplje, Serbia; 2 Faculty of Biology, University of Belgrade, Studentski trg 16, 11158 Belgrade, Serbia; 3 Faculty of Agriculture, University of Belgrade, Nemanjina 6, 11080 Belgrade, Serbia; 4 DIL German Institute of Food Technologies, Prof.-v.-Klitzing-Str. 7, 49610 Quakenbrueck, Germany

**Keywords:** bacteriobiota, “Pirot ‘ironed’ sausage”, chemical analysis, colorimetric characteristics, textural parameters, metabarcoding analysis, traditional manufacturing

## Abstract

“Pirot ‘ironed’ sausage“ (Pis) is a traditional, fermented sausage, made from different types of meat (beef and chevon), without additives or starter cultures. The physical–chemical properties (pH, water activity, fats, moisture, and protein contents) were examined in the initial meat batter stuffing and during ripening. Total bacterial diversity was examined at different time points using both culturable (traditional) and non-culturable (NGS sequencing) approaches. During the ripening, a decrease in pH value, a_w_, and moisture content was observed, as well as an increase in protein and fat content. At least a two-fold significant decrease was noted for colorimetric values during the ripening period. The dominance of *Proteobacteria* and *Firmicutes* was observed in the non-culturable approach in all studied samples. During the ripening process, an increase in *Firmicutes* (from 33.5% to 63.5%) with a decrease in *Proteobacteria* (from 65.4% to 22.3%) was observed. The bacterial genera that were dominant throughout the ripening process were *Lactobacillus*, *Photobacterium*, *Leuconostoc*, *Weissella*, and *Lactococcus*, while *Carnobacterium*, *Brochothrix*, and *Acinetobacter* were found also, but in negligible abundance. Among the culturable bacteria, *Latilactobacillus sakei* (*Lactobacillus sakei*) and *Leuconostoc mesenteoides* were present in all stages of ripening.

## 1. Introduction

Fermentation is one of the oldest methods of food processing and preservation. Fermented meat products are produced and consumed all over the world and represent one of the most important types of food. The use of fermentation processes changes the basic properties of the raw materials used, gives the product characteristic sensory properties, and makes it microbiologically safe and durable [1]. The fermentation of meat products is a complex biological process accelerated by the metabolic activity of desirable microorganisms in the presence of a large number of competitors or strains with synergistic activity [2]. Fermented sausages are dried, sometimes smoked, products made from various meats and firm, finely chopped fatty tissue, with the addition of salt, spices, sugar, approved additives, and starter cultures [3]. They are usually made from beef and pork, and the differences between them are due to the texture of the meat, but also to the presence of different species and genera of lactic acid bacteria (LAB), the degree of drying, spices, and the content of salt and sugar [4]. In general, they can be defined as raw sausages made from spiced cured meat that is stuffed into an intestine casing and left to ferment and mature [5]. With the process of ripening or aging, these sausages acquire their special quality, and also become microbiologically and chemically stable [6]. Although fermented sausages are considered microbiologically stable and safe products, the safety of these products may be compromised in cases of the high initial contamination of the raw product or insufficient control during the production process. Starter cultures represent a mixture of selected strains of beneficial microorganisms, and are added to products as an important tool to ensure the safety, uniformity, and lasting stability of a product [7]. They have no negative impact on the product, and improve its quality. Nowadays, there is a growing need for starter cultures, to obtain products with the highest possible quality [8]. Nitrite salts are additives that provide a stable red color to meat and inhibit the growth of microorganisms such as *Clostridium botulinum*, *Staphylococcus aureus*, and *Listeria monocytogenes*. They inhibit lipid oxidation and thus exhibit desirable antioxidant activity that contributes to the development of a pleasant odor and taste [9]. Currently, there is no known substance that could at least partially replace nitrites in meat products, especially considering their antibotulism effect. Under certain conditions, nitrites are involved in the formation of carcinogenic N-nitrosamines, which is why the use of nitrites and nitrates has been the subject of research for years, based on which it was concluded that it is not advisable to remove nitrites and nitrates from use, as this would lead to major problems such as botulism, low sustainability, and poor product quality [10]. The presence of *Brochothrix thermosphacta* and *Enterococcus faecium* in the first days of ripening of Fabriano-like sausage without the addition of nitrites/nitrates was demonstrated. However, the study showed that the higher the nitrite/nitrate concentration, the fewer species such as *Pseudomonas* spp., *Serratia liquefaciens*, and *Staphylococcus* spp., were present, while samples without nitrite/nitrate addition always had higher numbers of *Staphylococcus* spp. and *Pediococcus pentosaceus*. The data obtained clearly indicate the dominance of *Lactobacillus sakei* at all stages of sausage ripening, suggesting that the addition of nitrates/nitrites has no effect on the development of lactobacilli [11].

In Serbia, however, many fermented sausages are still made traditionally, without the addition of starter cultures. Sausages with long ripening periods, produced by traditional methods, taste better and are of higher quality than industrially produced sausages and defined starter cultures, which is due to the structure and metabolic activity of autochthonous cultures [8]. Nevertheless, the strict hygiene, the selection of the meat and the environmental conditions, as well as the addition of nitrite salts in traditional production can have a great influence on the final characteristics and safe consumption. In traditionally produced sausages, the addition of salt and other spices is fundamental to increasing the number of beneficial bacteria (such as lactic acid bacteria—LAB), which can lower the pH of the environment and in this way reduce the number of pathogenic bacteria. LABs are essential for the production process of various types of sausages, because under certain conditions, to which they are specially adapted, they initiate the fermentation process [8]. The microbial profile of fermented sausages mostly consists of LAB, above all, of the genera *Lactobacillus*, *Leuconostoc*, and *Lactococcus*, as well as related species of the phylum *Firmicutes* [12]. An important role of these bacteria is related to their metabolic activity, namely with the conversion of carbohydrates into organic acids and other metabolites. LABs are, actually, a heterogenic group of Gram-positive, acid-tolerant bacteria, some of which have a very important role in biotechnology and the food industry preservation [13]. The decrease in pH is of crucial importance because it contributes to the appearance of a characteristic taste, color, and aroma, as well as to the microbiological stability of a product [14]. Apart from that, LABs also possess characteristics, such as the production of bacteriocins, which can contribute to the quality of meat products [15]. Studies related to fermented sausages pointed out the presence of groups of bacteria as the most abundant ones: lactobacilli and micrococci and coagulase-negative staphylococci or catalase-positive cocci in general [15,16]. The members of the genus *Lactobacillus* most commonly isolated from dried sausages according to old taxonomy were *L. sakei*, *L. curvatus*, and *L. plantarum* [17]. The identification of the different species of lactobacilli that dominate the microbiome of fermented sausages is an important step in the search for new starter cultures for meat fermentation. Recently, a group of authors proposed a major reclassification of the old genus *Lactobacillus* into 23 new genera, including *Latilactobacillus* (*Latilactobacillus sakei*, *Latilactobacillus curvatus*, *Latilactobacillus graminis*, etc.), *Lactiplantibacillus* (*Lactiplantibacillus plantarum*), *Dellaglioa* (*Latilactobacillus algidus*), and others [18].

One of the typical products in Serbia is the flat so-called “Pirot ‘ironed’ sausage” (Pis), an unprocessed product, without preservatives, heat treatment or smoke. It is a famous specialty traditionally made of high-quality meat mixed with natural spices, without additives or starter cultures. Several carefully selected pieces of fat-free goat, lamb, beef, and even horse or donkey meat are used to make Pis [19]. Each of the ingredients plays a specific role in the process of the sausage ripening. Clean air, uncontaminated soil, and a good climate are important features of the area of the Stara Planina Mountain, in whose valley the town of Pirot is located, but the herbs that the animals eat also play an important role in the unique quality of the sausage. The sausages are ’ironed’ several times a week with a glass bottle to increase their surface and obtain a horseshoe shape. In this way, excess water and oxygen are released, which extends the shelf life of the final product.

This sausage is very interesting for research to discover variations and shifts of bacterial communities in the different phases of drying and ripening, while describing the physicochemical and technological parameters. To bring Pis to the market as a unique and high-quality product, it was necessary to analyze its microbiome. As far as we know, this is the first study describing bacteriobiota, and the technological parameters of this important traditionally produced sausage with a long history, during the aging process in a controlled environment. The differences in the drying method and duration of the drying process, the use of an unusual meat combination, the need for comparison between controlled and natural production, and the existence of numerous producers on the market provide much research material for the near future.

## 2. Materials and Methods

### 2.1. “Pirot ‘Ironed’ Sausages”

A total of 21 “Pirot ‘ironed’ sausages” (Pis) manufactured by the same producer (Boem) in a drying chamber located in the city of Pirot in southern Serbia (43.1557° N, 22.5857° E) were used for this study (three sausages per sampling time from one batch). The Pis were made in November and December 2018 in smaller quantities out of the most valuable meat cuts of beef and goat, i.e., chevon (in a ratio 50:50), previously cleaned of visible fat. The meat was mixed by adding only natural spices including sodium chloride salt, garlic, hot ground pepper and black pepper. The meat batter was stuffed into a thin 40 mm diameter bovine small intestine, and a horseshoe shape was formed. The sausages were dried at the temperature from 0 °C to 5 °C, for up to 28 days in a drying chamber, with RH values between 75–85% without airflow. During the drying period, the sausages were pressed three times a week with a glass bottle.

To test the physical–chemical and technological parameters during the ripening process of Pis, the samples were collected in five time periods (0, 7th, 14th, 21st, and 28th day with three sausages per sample time point and analyzed separately), while the successions of non-culturable and culturable bacterial communities were monitored during six time periods (0 (sample Z2), 2nd (sample Z3), 7th (sample Z4), 10th (sample Z5), 14th (sample Z6), and 21st (sample Z7) day with three sausages per sample time point pooled into one sample on the end), including a sample of small bovine intestine (sample Z1). The samples were vacuum-packed during all time-points collected and then transported to the laboratory in a refrigerated box for further analyses.

### 2.2. Metabarcoding Analysis

#### 2.2.1. DNA Extraction, Library Preparation, and NGS Sequencing

The samples were taken from randomly chosen parts of sausages using the dynamics described within Section 2.1, by making small cuts in several places with a sterile scalpel. All parts collected from three sausages were pooled and mixed into one sample. The DNA extraction was performed using the Zymo BIOMICS DNA Mini Kit (Zymo Research, Irvine, CA, USA) from a minimum of 200 mg/pooled sample from each time point, according to the manufacturer’s protocol. The concentration of isolated DNA was measured using a Qubit Fluorometric Quantitation device (Qubit 4 Fluorometer, Invitrogen, Carlsbad, CA, USA). Amplicon sequencing was performed using a 2 × 300 bp paired-end sequencer on a MiSeq sequencer according to the manufacturer’s instructions (Illumina, San Diego, CA, USA) at a commercial sequencing service (FISABIO, Valencia, Spain). To identify bacterial communities, the V3-V4 region of the 16S rRNA gene was amplified with defined forward (5′-CCTAGCGGGNGGCWGCAG-3′) and reverse (5′-GACTACHVGGGTATCTAATCC-3′) primers [20].

#### 2.2.2. Raw Data Processing and Taxonomy Annotation

A sequence quality assessment was performed using the DADA2 R package [21]. Primer trimming was performed with Bbduk [22] using the same primers as during sequencing, according to the specified functions: ktrim = l (trimming at the left end), hdist = 1 (Hamming distance), mm = f (ignoring the middle base of a kmer), with k-measure length 15. All forward reads were trimmed on 250 bp length, while reverse were trimmed on 200 bp together with all reads whose Phred score was lower than 6. All sequences that had more than four for forward and the reverse strand estimated errors (calculated as the sum(10^(−Q/10)), where Q is the Phred score) were excluded (argument: maxEE = c(3,2)), as well as all reads with undefined bases (N) and sequences that were shorter than 50 bp. A sequence merger was performed with a minimum overlap of 20 bases without mismatches. Chimeric sequences were removed using default parameters in the DaDa2 R package, and all sequences shorter than 400 bp and longer than 429 bp were also eliminated. A taxonomic annotation of 16S rRNA sequences was performed using the SILVA132 database (https://zenodo.org/record/1172783, accessed on 21 August 2021). An RDP Naïve Bayesian Classifier [23] with default options as implemented in the DADA2 package was used to classify taxonomy up to the OTU level. The homology of the identified OTUs was additionally annotated based on the BLAST*n* best bit score (accessed on 25 August 2020) in the NCBI 16S database and the first Top5 hits with the closest species taxonomy were used for analysis using the Joint Genome Project Taxonomy server (https://taxonomy.jgi-psf.org/, accessed on 25 August 2020).

#### 2.2.3. Statistical Analyses

Alpha diversity was estimated using the Phyloseq R program [24] at all taxonomic levels up to OTU. Alpha diversity indices in terms of species richness ACE, Chao1, and several observed species (OBS) were used. Diversity is shown through the Shannon, Simpson, and Fisher indices. In the beginning, the diversity between different samples (beta diversity) was estimated at the OTU level and determined using Double Principle Coordinate Analysis—DPCoA [25]. Sequence alignment was performed using the DECIPHER::AlignSeqs with default arguments [26]. This alignment was used to create a neighbor joining tree via phangorn package 2.5.5 [27]. A dendrogram was rooted according to midpoint (https://www.mun.ca/biology/scarr/Panda_midpoint_rooting.html, accessed on 30 August 2020). However, a variance stabilizing transformation was implemented in the DESeq2 package, and the multidimensional scaling transformation (MDS) was performed using weighted UniFrac distances [28]. Before calculating distances, samples were rarefied to even depth (according to the sample with the lowest read count), and transformed with a log(1 + x) transformation.

### 2.3. Culturable Bacteriobiota

The same sausage sample parts used for the non-culturable approach were taken for the growth of culturable bacteriobiota using the same dynamics described within Section 2.1. All parts collected from the three sausages were pooled and mixed into one sample. Ten grams of the pooled samples from each ripening point were sampled and immersed into 90 mL of saline solution with the addition of 0.9 g of peptone, shaken for 20 min, and serially diluted [19]. Dilutions were plated on de Man, Rogosa and Sharpe (MRS) agar, Luria–Bertani agar (LA), and Plate Count Agar (PCA), and growth was monitored for a minimum of 48 h at 30 °C under microaerophilic conditions.

Genomic DNA of each isolate was isolated using the protocol described in Dimkić et al. [29]. Molecular identification of the isolates was completed by amplification of the 16S rRNA gene with the universal 27F (5′-GAGAGTTTGATCCTGGCTCAG-3′) and 1523R (5′-AGGAGGTGATCCAGCCG-3′) primers. The PCR reaction mixture with a total volume of 25 µL per sample contained FastGene Taq 2x Ready Mix (12.5 µL, Nippon Genetics, Germany), 1 µL of each primer, 9 µL of PCR water, and 1.5 µL of DNA sample. The PCR reaction was performed in 33 cycles with primer hybridization at 50 °C for 1 min. Amplicons were purified with a QIAquick PCR Purification and Gel Extraction KIT/250 (QIAGEN GmbH, Hilden, Germany) purification kit and sent to a commercial service for Sanger sequencing (Macrogen sequencing service, Amsterdam, the Netherlands).

#### Phylogenetic Analysis

The sequences thus obtained were searched for homology with previously sequenced genes in the GenBank database, using the National Center for Biotechnology Information’s BLAST search program for 16S rRNA databases. To secure taxonomic relevance, the most closely related sequences of referent strains were used for phylogenetic analyses. All sequences were aligned using ClustalW multiple sequence alignment implemented in BioEdit free program ver. 7.0.5.3, and phylogenetic trees were constructed in MEGA X using the neighbor-joining method based on a pair-wise distance matrix with the Kimura two-parameter nucleotide substitution model.

### 2.4. Physical-Chemical and Technological Parameters during the Ripening

Preparation for the physical–chemical and technology parameters was performed and collected using the dynamics described within Section 2.1. For chemical analysis, all samples were homogenized with a chopper (Blixer 2, Robot Coupe, France), and pH, water activity (a_w_), percentages of moisture (ISO 1442:1997), total protein (ISO 937:1978), and fat (ISO 1444:1996) content tests were performed as described earlier [30]. Triplicate determinations of the chemical parameters were performed on each tested sausage. The colorimetric characteristics (L*—lightness; a*—redness; b*—yellowness) of all samples of the surface color of the sausage with and without casing, and cross-sectional surface were measured using the Computer Vision System and procedure [31], while the equipment was calibrated according to the procedure explained earlier by Tomasevic et al. [32]. Except for the surface color of the sausage without casing (only on the 14th, 21st, and 28th day of ripening—the first two points (0 and 7th day) could not be performed because the casing could not be removed from the raw sausages), all tests were performed throughout the maturation process. Textural characteristics were carried out using Warner–Bratzler Shear Force (WBSF) test [33]. The Warner–Bratzler ‘V’ slot blade was attached to the universal testing machine (TA.XT Stable Micro System Corporation, Godalming, UK) and the shearing speed was 1.50 mm/s. The load cell capacity used in this study was 50 kg. The sample diameter was about 10 mm. Two parameters were measured: the maximum shear force (N) and work of shearing (N × sec)—the total shear work necessary to cut through the sample. At least eight technical replicates were used for each sample and measurements were conducted in triplicate.

#### Statistical Processing Analysis

The obtained values for all parameters, supported by the Kruskal–Wallis test for normality, were used for testing variance significance among the samples during ripening by Tukey’s HSD post hoc test (One-way ANOVA). Statistical significance applied in all tests was *p* < 0.05. Statistical analysis was performed using the software program IBM SPSS Statistics v.23 (IBM SPSS Inc., Armonk, NY, USA).

## 3. Results

### 3.1. Total Bacteriobiota Diversity during the Ripening

A total of 1,053,569 raw sequences were obtained from the 16S library sequencing (from 107,113 to 189,000 per sample). After primer removal, denoising, quality filtering, length trims, and chimera removal, the number of reads ranged from 86,161 up to 146,679 (Table S1). The mean length of obtained OTU sequences was 415, and 1035 OTUs in total were scored. Based on the alpha rarefaction curves, the diversity indices were estimated after rarefaction to a uniform depth corresponding to the sample with the lowest number of reads (sample Z4), because there were obvious differences in reads between samples.

#### 3.1.1. The Analysis of Alpha and Beta Diversities

The diversity and richness of bacterial communities were investigated at the different time points of the sampled material during the ripening process on four taxonomic levels (the phylum, family, genus, and the OTU), as presented in Table 1. The indexes which denote the richness of bacterial species (OBS, Chao1, and ACE) showed that compared to all the studied taxonomic hierarchies, except at the phylum level, the highest richness of species was noted for the bovine small intestine.

As far as indexes that indicate bacterial diversity are concerned, the OTU indices (the Shannon, Simpson, and Fisher) indicated a high level of bacterial diversity within the intestine sample, while for the samples collected during the ripening of sausages, a lower, but a relatively good diversity was noted. At the level of the phylum, there was no difference, and the expected lowest level of diversity was noted. However, the differences between samples during ripening were not observed at all taxa levels.

**Figure 1:**
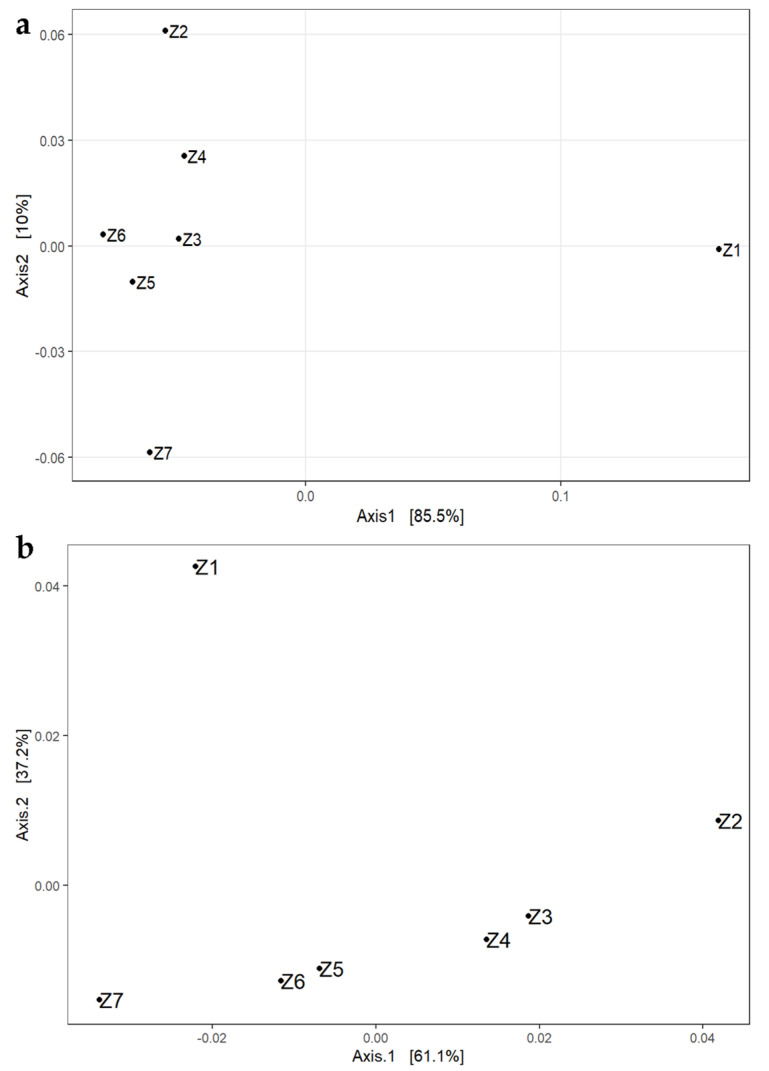
The DPCoA (**a**) and MDS (**b**) plots at the OTU level showing the variability between samples. Intestine (sample Z1); days of ripening: 0 (sample Z2), 2nd (sample Z3), 7th (sample Z4), 10th (sample Z5), 14th (sample Z6), and 21st (sample Z7).

For the visualization of beta diversity, the initial analysis was performed with Bray–Curtis dissimilarities, and samples were rarefied to even depth on the OTU level (Figure 1a).

Based on the results, it can be concluded that the samples from the ripening process are joined together, and fully distant according to the first axis (85.5%) from the sample of the intestine. The samples from the beginning and the end of the ripening process Z2 and Z7 showed the furthermost distance from the other samples obtained during ripening. However, a more suitable stabilization of variance was carried out later, and results were obtained from multidimensional scaling (MDS or PCoA) based on the used weighted UniFrac distances (Figure 1b). A similar distribution is shown among samples. Moreover, samples Z3 and Z4, as well as Z5 and Z6, were closely related to the other samples during the ripening process. Values on the axes were scaled based on their values. The greatest diversity was noted for the intestine sample (sample Z1) explaining 61.1% and 37.2% of the variability, respectively, according to the axes values.

#### 3.1.2. A Taxonomic Annotation of the Total Bacteriobiota

The dominance of the phyla *Proteobacteria* and *Firmicutes* were noted for all the studied samples, including the intestine sample (37.9%, and 59.1%, respectively). During the ripening process, almost double the increment of *Firmicutes* (from 33.5% to 63.5%), with a simultaneous decrement of *Proteobacteria* (from 65.4% to 22.3%), was characteristic, including the unknown *Cyanobacteria*, later confirmed as a chloroplast, and removed from the further analyses. The remaining phyla, such as *Bacteroidetes*, *Epsilonbacteraeota*, and *Actinobacteria* were also found in the intestine-pooled sample. The phyla with a considerably lower relative abundance (RA) were *WPS-2* and *Fusobacteria*.

**Figure 2:**
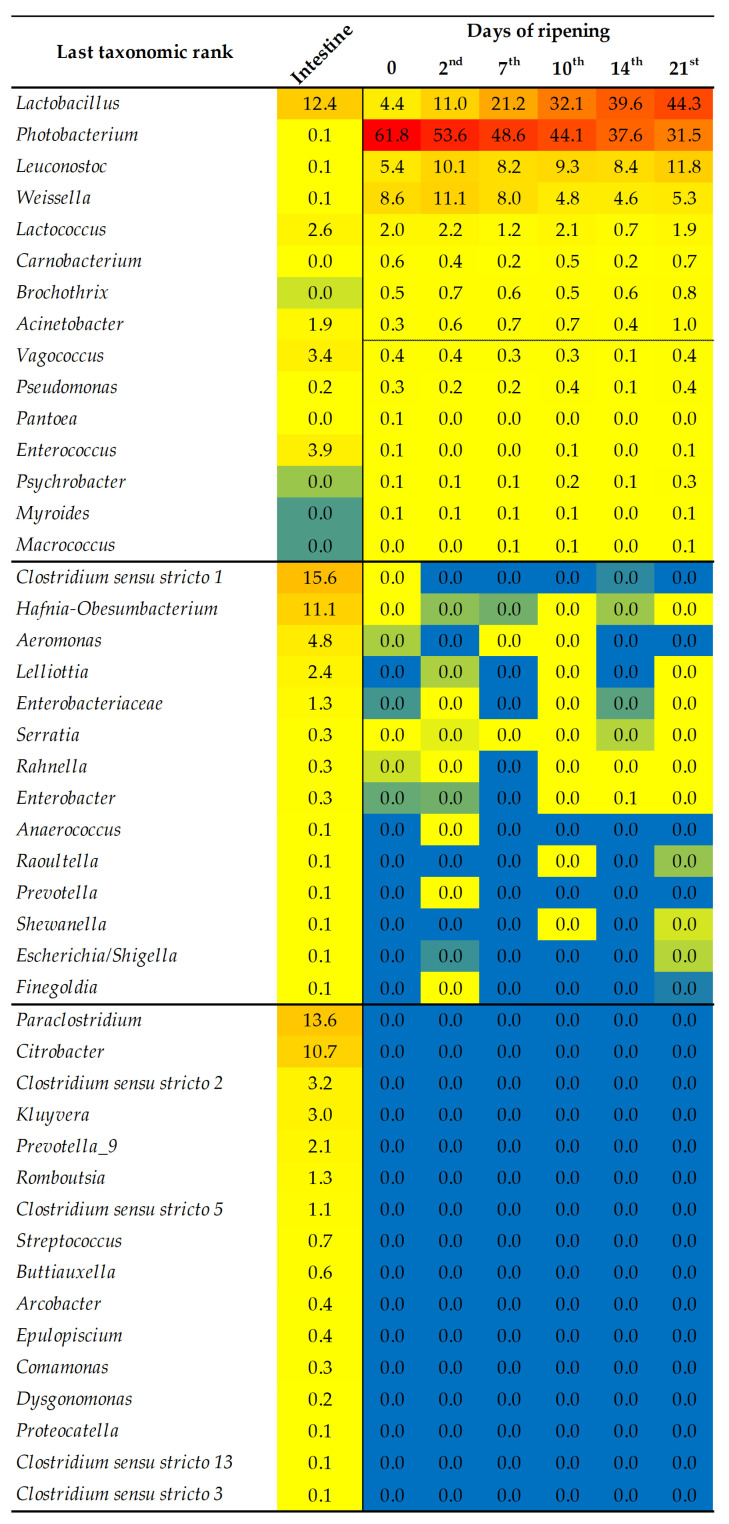
Relative abundance of bacterial taxa at the genus taxonomic rank according to the SILVA 132 database. Different colors indicate low or high abundance (blue, yellow, and red colors—lowest, medium, and highest RA).

The bacterial taxa which dominated the entire ripening process and which were found in a greater or lesser relative abundance (RA) according to SILVA 132 database belonged to the main genera of the *Lactobacillus*, *Photobacterium*, *Leuconostoc*, *Weissella*, and *Lactococcus*. Contrary to that, *Carnobacterium*, *Brochothrix*, and *Acinetobacter* were also found but in a negligible RA compared to the previous genera (Figure 2). Community shifts are very well represented by a *Lactobacillus* increasing trend from the beginning of the ripening, while *Photobacterium* and *Weissella* had a negative correlation and decrement of abundance. *Leuconostoc* and other listed genera were detected more or less in the same abundance throughout the ripening process.

The most prevalent taxa linked to the variety of intestine communities (which could be found to a negligible lesser extent during the ripening of sausages) were *Clostridium sensu stricto 1*, *Lactobacillus*, *Hafnia-Obesumbacterium*, *Aeromonas*, *Enterococcus*, *Vagococcus*, *Lactococcus*, *Lelliottia*, *Acinetobacter*, and unknown representatives of the family *Enterobacteriaceae*. Additionally, the taxa exclusively found in the bovine small intestine niche in relatively higher RA were *Paraclostridium*, *Citrobacter*, *Clostridium sensu stricto 2*, *Kluyvera*, *Prevotella 9*, *Romboutsia*, *Clostridium sensu stricto 5*, *Streptococcus*, and *Buttiauxella*. All other taxa were found in RA of lower than 0.5% and did not have a significant contribution to the ripening of the examined samples. Interestingly, according to the Bray–Curtis dissimilarities at the OTU level, grouping between samples throughout ripening showed the variability within the samples and close relatedness of the Z6 and Z2 samples (Figure S1).

After taxonomy annotation based on OTU homology with the BLASTn best bit score in the NCBI 16S database, the most diverse taxa at the species level of the main genera sequences within the ripening process were obtained (Table S2). Although the sequence lengths on average were more than 400 bp, some distribution of the species can be observed. After evaluation, the number of detected sequences within *Photobacterium* belongs to *P. carnosum*, *P. phosphoreum*, *P. iliopiscarium*, and *P. piscicola*, while the most represented *Lactobacillus* species according to the new reclassification were *Latilactobacillus* (*L. sakei*, and *L. graminis*) and *Dellaglioa algidus* (basonym *Latilactobacillus algidus*) (among 11 different species scored as shown in Table S2).

Six species of *Leuconostoc* (*Ln. gelidum*, *Ln. mesenteroides*, *Ln. pseudomesenteroides*, *Ln. carnosum*, *Ln. holzapfelii*, and *Ln. lactis*) and three species of *Weissella* (*W. fabalis*, *W. koreensis*, and *W. cibaria*) were revealed. Among *Lactococcus* and *Carnobacterium* sequences, four (*Lc. raffinolactis*, *Lc. lactis*, *Lc. garvieae*, and *Lc. piscium*) and three (*C. divergens*, *C. gallinarum*, and *C. jeotgali*) different species were annotated, respectively. Only *Brochothrix thermosphacta* was detected, while 12 different species belonged to the *Acinetobacter* genus, as well.

### 3.2. An Analysis of the Culturable Bacterial Communities

Following the growth on different media and morphology of axenic bacterial cultures, 157 isolates were obtained (the list of all isolates and species names are presented in Table S3). However, only 32 different and unique species were determined by analyzing the 16S rRNA sequences (Table 2).

**Figure 3:**
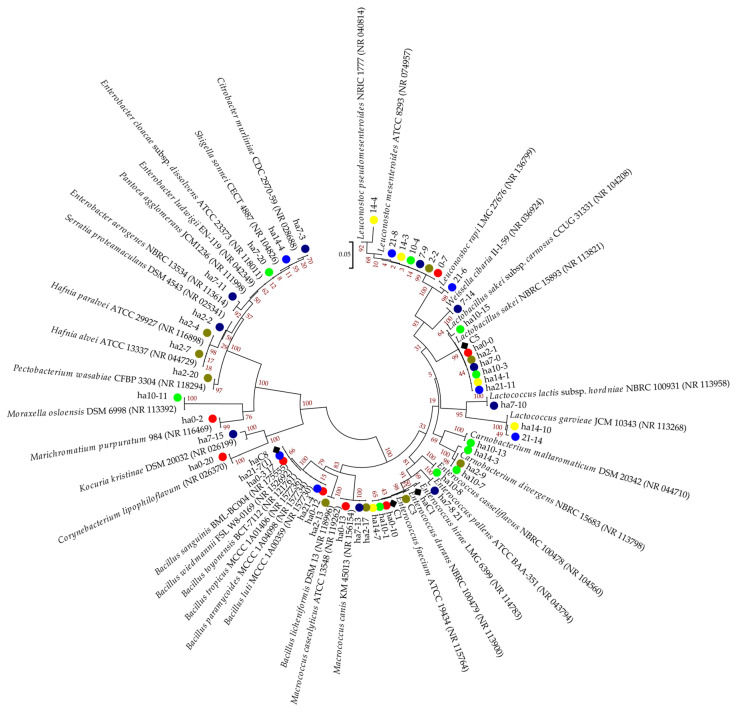
Neighbor-joining phylogenetic tree based on a pair-wise distance matrix with the Kimura two-parameter nucleotide substitution model, showing the relationship of the 16S rRNA sequences among obtained isolates. The topology of the trees was evaluated by the bootstrap resampling method with 1000 replicates. (♦ intestine isolates; ● 0 day; ● 2nd day; ● 7th day; ● 10th day; ● 14th day; ● 21st day of ripening).

The relationships of the 16S rRNA sequences among the obtained representative isolates from all samples are shown on the dendrogram (Figure 3).

The lowest number of isolates was obtained from the bovine small intestine sample and most of the isolates belong to *Enterococcus* species (*E. faecium, E. hirae*, and *E. durans*). *Lactobacillus sakei* was the only species common to all samples, while *Leuconostoc mesenteroides* dominated the number of isolates during the ripening process (Table S3), and was not isolated from the intestine (Figure S2). *Leuconostoc pseudomesenteroides* and *Ln. rapi* were also isolated at the end of the ripening process. Greater diversity was noted at the beginning of the ripening process and slightly decreased at the end of the monitored period. The samples Z3, Z5, and Z6 shared the presence of *Carnobacterium divergens*. Z3 and Z4 were characterized by the appearance of the *Macrococcus canis*, while *Lactococcus garvieae* were present on the 14th and 21st day of ripening. Additionally, another representative of *Lactococcus* (*L. lactis*) was found within sample Z4, which was the most abundant with seven unique species, including, among others, *Enterococcus pallens*, *Weissella cibaria*, *Citrobacter murliniae*, and *Enterobacter aerogenes*. Furthermore, samples collected during the 2nd and 10th day were also populated with unique species such as *Pectobacterium wasabiae*, *Hafnia alvei*, *H. paralvei*, *Serratia proteamaculans*, *Bacillus licheniformis*, *Carnobacterium maltaromaticum*, *Moraxella osloensis*, and *Enterococcus casseliflavus*.

### 3.3. Physical-Chemical and Technological Parameters during the Ripening

**Figure 4:**
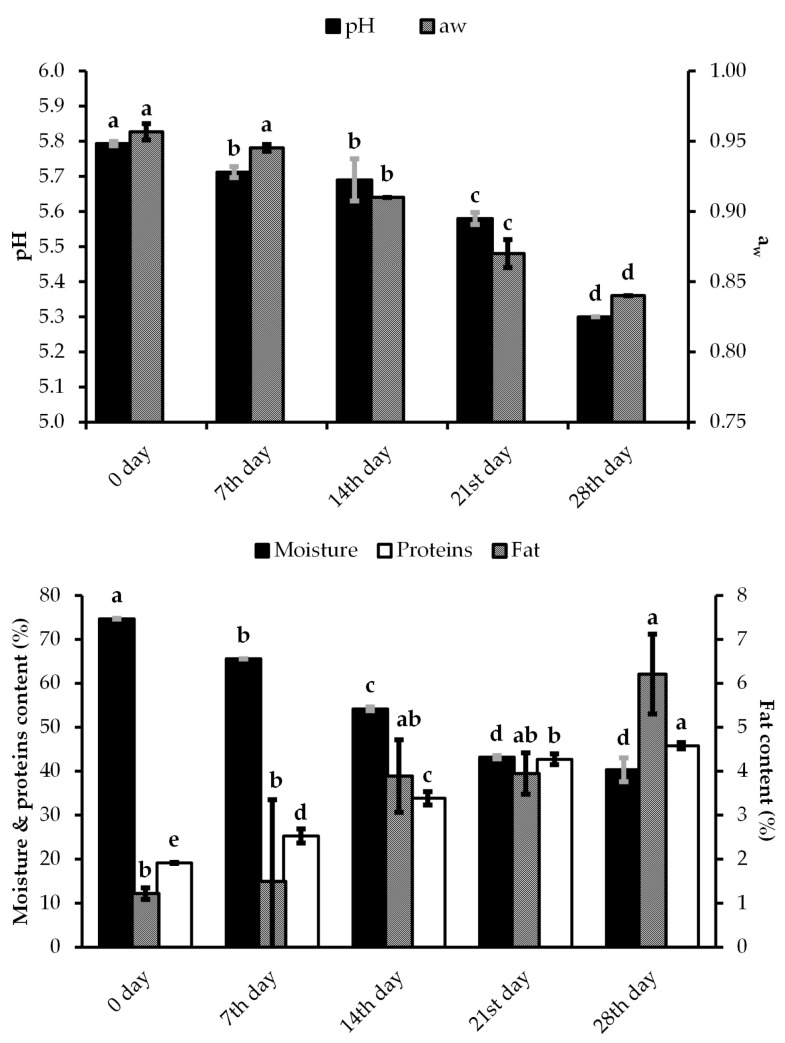
Physical–chemical parameters during ripening of “Pirot ‘ironed’ sausage” (mean ± st. deviation). Values within the same parameters during ripening followed by different letters are significantly different (*p* < 0.05), according to Tukey’s HSD test.

The pH values significantly dropped throughout ripening, and they were positively correlated with a water activity and average moisture content (Figure 4).

The lowest pH, a_w_, and average moisture content values were characteristic for the 28th day of ripening with 5.30, 0.84, and 40%, respectively. According to the mean difference between the time points of ripening, it is obvious that pronounced statistical significance was observed between all samples, except between the 7th and 14th day for pH, i.e., 0 and 7th day for a_w_ values, as well as the 21st and 28th day for moisture content (Table S4). The average initial fat content of sausages was lower and moderate for total protein content. The decrease in moisture during the ripening significantly contributed to the increase in fat (six-fold more) and protein (2.5-fold more) contents with maximum values measured on the 28th day. Only fat content was statistically significant between days 0 and 28 and between days 7 and 28, whereas protein content was significant throughout the ripening period.

**Figure 5:**
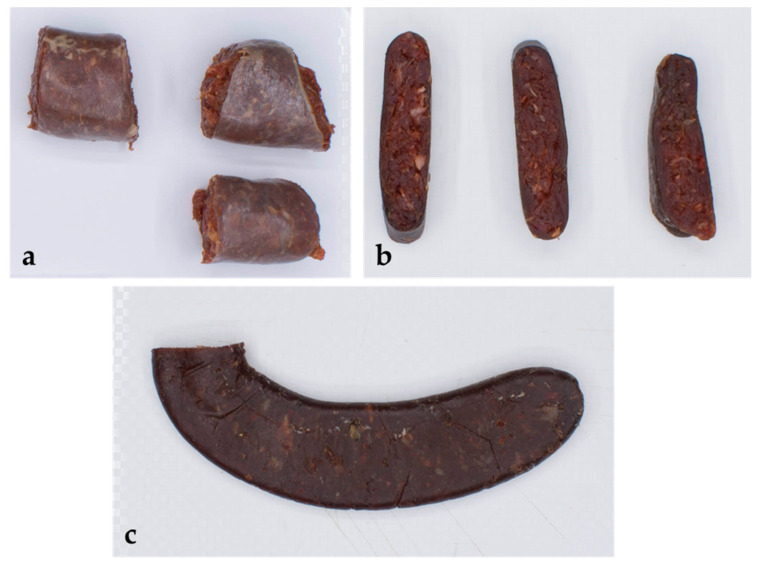
The colorimetric characteristics measured of the surface color of the sausage with and without casing (**a**,**c**) and cross-sectional surface (**b**).

The colorimetric characteristics (L*—lightness; a*—redness; b*—yellowness) of the surface color of all the sausage samples with and without casing, and cross-sectional surface were measured (Figure 5).

During the measurement of colorimetric characteristics (L*, a*, and b*), the parameters of the sausages without casing were not followed due to the impossibility of removing it from raw sausages. The initial L* value of the surface color of the sausage with casing decreased significantly till the end of the measurement, while the redness value also decreased by double. Furthermore, a significant decrease was noted for L* and a* values during ripening for cross-sectional samples and sausages without casing. The color difference was even more noticeable for the b* values with the negative score at the end of the ripening process for the sausage with and without casing. The measured values indicated that the color of the sausage became blue as the values were negative. Both, L* and a* values significantly decreased two-fold during the ripening period. According to the mean difference between the time points of ripening, it is obvious that strong statistical significance was achieved for the a* and b* values observed among all samples for cross-sectional samples and sausages with casing (mostly at *p* < 0.001), except between the 14th and 21st day, i.e., 21st and 28th day for a*; 0 and 7th, 7th and 14th, 14th and 28th, as well as 21st and 28th day for sausages with (Table S4). Both a* and b* values between samples of the 0 and 7th day for cross-sectional samples were not statistically different. The Pis textural characteristics were expressed through the firmness and toughness, which significantly gradually increased during ripening (Table 3).

## 4. Discussion

A very specific method of production, without additives, without processing, as well as a very specific climatic region make the “Pirot ‘ironed’ sausage” (Pis) a very special product, and knowledge of its microbiome can help to understand the unique characteristics of this traditional Serbian brand. Studying the ecology of fermented sausages is essential to understanding the physical and chemical changes that occur during fermentation and ripening. Furthermore, the specific bacteriobiota of the sausages is responsible for the characteristics of the final product. Studies on sausages produced by fermentation were carried out on typical products from Greece, Spain, Turkey, and Italy. The microorganisms present in the initial stages of the ripening process are mostly derived from raw products, spices, and the environment [34]. The number of lactic acid bacteria (LAB) at the beginning of the fermentation process can vary greatly. These microorganisms multiply very rapidly and predominate during the first days of fermentation. This number remains stable during ripening, although the survival rate may decrease during the dehydration phase [8,34,35].

The microbiota present contributes strongly to fermentation, especially during the ripening of sausages. Bacterial diversity can be studied using culture-dependent and independent methods. However, for a more accurate characterization of microbial diversity, high-throughput sequencing can be used to detect the complex communities and microorganisms in low abundance [36]. In previous studies, most of the sequence reads of dry fermented sausages were found to belong to *Proteobacteria* and *Firmicutes*, which accounted for more than 90% of the total abundance, which was also the case in our study [36,37]. Moreover, it was reported that the total proportion of *Proteobacteria* and *Firmicutes* was higher in long sausages than in round sausages [36]. The bacterial genera that dominated the ripening process of the Pis included *Lactobacillus* (recently reclassified into 23 new genera [18]), *Photobacterium*, *Leuconostoc, Weissella*, and *Lactococcus*. Bacterial diversity was high and the succession of the bacterial community toward *Lactobacillus* increased, while *Photobacterium* and *Weissella* decreased. Similar results were confirmed by Wang et al. [38], where *Lactobacillus* increased to RA 56% and became the dominant genus at the end of ripening, as was the case in our study. We have previously confirmed the dominance of *L. sakei* in Pis [19], and other studies have shown that this species is best adapted to fermented sausages and is one of the most frequently detected psychrotrophic lactobacilli [34,39]. Beef products were mainly represented by *L. curvatus*, *L. sakei*, and *Leuconostoc* spp. [40]. Such dominance of *L. sakei* certainly deserves in-depth investigations of its selective development during meat storage and ripening. Moreover, *Leuconostoc mesenteroides* was also confirmed for Pis products from different manufacturers in our previous study [19]. The role played by *Leuconostoc* in fermented sausages has not been studied in detail and its presence is rather controversial. *Leuconostoc*, a heterofermentative genus, produces CO_2_ that forms holes in meat products, which are considered undesirable characteristics [41]. On the other hand, they contribute to the characteristic flavor and aroma of fermented sausages, through the use of citrates and the production of acetic acid, acetaldehyde, diacetyl, and ethanol [42]. *Leuconostoc* spp. are usually absent or present in lower RA in dry fermented sausages [43] and are rarely part of the dominant microbiota [44]. The community of LAB in Ventricina, a traditional fermented sausage from central Italy, included *Ln. mesenteroides* and *L. sakei* (the most abundant species) as a common microbiota in traditional sausage [45]. *Lactococcus* is not always found in dry fermented sausages, but the most frequently isolated strain is *Lactococcus lactis* [44]. In our study, we isolated *Lactococcus garvieae* in addition to *Lactococcus lactis* subsp. *hordniae*, which was confirmed by metabarcoding data. Apart from these species, *Lactococcus raffinolactis* was the predominant species within the OTUs. Earlier it was shown that *Lc*. *raffinolactis* was predominant in the bacterial diversity of traditional dry sausages from northeastern China, while *Lc. lactis* had a positive correlation with the volatile aromatic compounds involved in flavor and aroma formation [46]. The decline of *Lactococcus* during fermentation is due to its sensitivity to lower pH values and higher concentrations of salt [47].

The relative abundance of potentially spoiling and harmful bacteria (except *Photobacterium*), such as *Acinetobacter*, *Aeromonas*, *Pseudomonas*, *Brochothrix*, *Psychrobacter*, *Serratia*, *Rahnella*, *Shewanella*, *Escherichia*, *Moraxella*, *Hafnia*, etc., was found to be in negligible percentages throughout the ripening process. However, some of them were obtained by the culturable approach but with only a few isolates. Moreover, the growth of these bacteria in fermented sausage may be due to contamination during the handling and storage of the product [48]. According to NCBI results, only *B. thermosphacta* was found as a species in our samples, which is known to be a natural contaminant in food and inevitably enters meat processing plants. However, *Brochothrix* spp. are less of a problem for fresh meat, which has a pH of 5.3 or 5.5 and is often stored at lower temperatures during distribution and retail display [49]. In contrast, *Photobacterium* spp., already proven as ubiquitous psychrophilic bacteria on all common meats, including sausages [50], were found in higher RA in our samples, especially at the beginning of ripening. BLASTn best bit scores showed the presence of four *Photobacterium* OTUs (*P. carnosum*, *P. phosphoreum*, *P. iliopiscarium*, and *P. piscicola*, with the first being the most abundant). *P. carnosum* was shown to be well adapted to meat-rich environments based on its growth and metabolic traits, as stated before [51]. *P. carnosum*, *P. phosphoreum*, and *P. iliopiscarium* were found in modified-atmosphere packages of untainted and spoiled meat [51]. Previously, *Photobacterium* was found to be relatively abundant in pork stored under air or vacuum and may play a greater role in meat spoilage than previously thought [52]. Previous studies have also shown that *Photobacterium* require a complex medium with a high NaCl content for growth, and even the use of a standard medium such as PCA was not sufficient to culture the isolates, although high-throughput sequencing revealed *Photobacterium* as the predominant microflora in pork sausages [53]. However, the same authors found that the relative abundance of *Photobacterium* decreased during storage, which was also the case in our study. Possibly, this fact at least indicates that *Photobacterium* was in a non-culturable state and thus had no effect on the fermentation of Pis sausage. Among other genera, *Photobacterium* was also found in Chinese-style sausages, which were probably of poor hygienic quality due to spontaneous fermentation [54]. It is also reported that *Photobacterium* was always isolated in 100% of beef samples [55]. In addition, the study by Li et al. [56] using a correlation network model showed that *Pediococcus* and *Lactococcus*, followed by *Enterobacter*, *Citrobacter*, and *Photobacterium* contributed the most to the formation of the volatile flavor of sausages. Therefore, a cultivation method for *Photobacterium* and its inclusion in the standard microbiological analyses of meat products is of the utmost importance for future work. The RA of all detected *Photobacterium* OTUs gradually decreased to a two-fold lower level at the end of ripening and was positively correlated with an increase in *Lactobacillus*. This was also confirmed in the study of Fuertes-Perez et al. [50], in which *L. sakei* was also an important species at the end of the ripening of Spanish-type chorizo. However, the effect of *Photobacterium* spp. on meat spoilage remains to be investigated in further studies. Although a gradual decline of *Weissella* from the *Leuconostocaceae* family was observed during ripening, *Lactobacillus* and *Weissella* are generally considered the most important LAB in fermented sausages [57]. Our results indicate the existence of three *Weissella* species (*W. fabalis, W. koreensis*, and *W. cibaria*). Previously, *W. cibaria*, *W. paramesenteroides*, *W. hellenica*, and *W. viridescens* were frequently detected in fermented sausages [58], while *W. koreensis* was conspicuous in kimchi fermentation [59]. *W. viridescens* and *W. confusa* were isolated from Turkish dry sausages [16] and *Weissella* was also detested in Bosnian Sudžuk [60]. *Weissella fabalis* was detected here for the first time in the ripening process of sausages, whereas it was previously isolated in the spontaneous fermentation of cocoa beans [61].

In addition, *Carnobacterium divergens* and *Carnobacterium maltaromaticum* were identified in our study. However, the presence of *Carnobacterium* in low numbers was recorded in analyzed fermented sausages from game meat [62], which is in agreement with our study. They are usually found in meat products and it has not yet been proven that they are human pathogens, but strains of *Carnobacterium* cause food spoilage, even at low temperatures [40]. For *C. divergens*, its predominance in raw meat, regardless of packaging conditions, has been pointed out, while for *C. maltaromaticum* it has been suggested that its actual role in the sensory spoilage of meat can be considered negligible [63]. In addition to the presence of *C. divergens* and *C. maltaromaticum*, psychrotrophic *L. sakei*, *L. curvatus*, *L. fuchuensis*, and *Leuconostoc* sp. were reported to be prevalent in cold-stored meat [64]. A previous study on the composition of an artisanal Serbian sausage (Petrovskă Klobăsa), whose manufacturing and drying process is similar to that of Pis production, confirmed the presence of *E. durans* and *E. casseliflavus* [44], which were also isolated in our study. In a previous study, the production of the antilisterial bacteriocin enterocin 416K1 was confirmed for *E. casseliflavus* isolated from Italian sausages [65]. Our study also confirmed the presence of two *Hafnia* species (*H. alvei* and *H. paralvei*). The commensal *Hafnia alvei* is used for fermentation in the dairy industry, and more recently as a probiotic in the production of dietary supplements [66]. We also isolated *Moraxella osloensis*, *Citrobacter murliniae*, *Macrococcus canis*, *Macrococcus caseolyticus*, *Marichromatium purpuratum*, *Serratia proteamaculans*, *Pantoea agglomerans*/*Enterobacter ludwigii*/*Enterobacter cloacae*, *Shigella sonnei*, and *Kocuria kristinae*, all bacteria not commonly isolated from fermented products. They are all classified as pathogenic or undesirable bacteria and probably occur due to poor hygienic storage conditions. However, some of them, such as *S. proteamaculans*, *H. alvei*, *H. paralvei*, and *Citrobacter* sp., have been found be present in pork, beef, and game meat during the first seven days of fermentation [67]. Interestingly, *M. purpuratum* was shown to be a normal component of natural casings derived from swine intestines [68]. Finally, *Pectobacterium wasabiae* from the Enterobacteriaceae family, a plant pathogen [69], has not been registered in any similar study on the ripening process of sausage.

We have already reported on the description of the chemical changes in Pis during ripening [30]. After initially higher values, a significant linear decrease in pH was observed, which is in agreement with the published literature data in comparison with other traditionally produced sausages [70,71,72,73]. A decrease in pH was also observed in the fermentation of Petrovac sausage, while a sharp increase in the total number of mesophilic bacteria and LABs was observed after two days of fermentation [44]. Ground pepper (paprika), one of the main ingredients, contains 9.6–13.2% sugar, which is fermented to lactic acid by the sausage microbiota during the initial stage of ripening [73]. Salgado et al. [74] state that the increase in pH compared to the lowest value reached during the fermentation process implies the end of the fermentation process and the beginning of the proteolytic and lipolytic processes, i.e., the beginning of the ripening process. The average pH of 5.3 in the final product is important for moisture release (drying), shelf life, and the formation of color, consistency, and aroma of the traditionally fermented sausage [73]. A rapid lowering of the pH value, as in our case, during fermentation, ensures the safety of the product by inactivating pathogens, which leads to greater stability and the extending of the shelf life of the product. Our results show a decrease in the water activity of Pis after ripening for 28 days, at a temperature of 0 °C to 5 °C. It is known that pathogenic bacteria cannot multiply at an a_w_ value of less than 0.95. Therefore, it can be concluded that Pis are a bacteriologically stable product even during ripening, which can be stored at room temperature [75]. The decrease in the water activity value from 0.96 (meat stuffing) to 0.84 (after 28 days of production) is mainly due to drying, i.e., the decrease in moisture content from an initial 74.72% to 40.32%. The average initial moisture content is higher than the previously determined [76], but it is identical to that of “Lemeški Kulen” [73]. This can be explained by the fact that only meat without additional fat is used for the production of Pis. Moreover, the moisture content in our work decreased faster during ripening than in sausages containing pork fat [71,76]. One of the reasons is the ironing of the sausages with a glass bottle, so the surface area to volume ratio is favorable for water evaporation. According to Fanco et al. [77], a decrease in moisture during maturation leads to an increase in fat and protein content, which is also confirmed by the results of our study. The protein content in the Pirot iron sausage is significantly higher compared to other traditionally produced sausages in Serbia [72]. The protein content was also higher in comparison with sausages made from horse meat, although horse meat has a higher protein content compared to beef [78]. In comparison with beef and goat Sudžuk sausage and goat Frankfurter sausage, the value of protein content is also higher [79,80]. The initial average value of the protein content is close to the value measured in Petrovac sausage [72]. The average initial fat content (1.22%) in the sausages was significantly lower compared to Petrovac and Srem sausages [72,81]. The fat content of the final products was lower than the fat content of Srem sausages (made from horse meat), or some traditional Spanish sausages [71,78,82,83].

In traditional fermented sausages, made without the addition of nitrate and nitrite salts, the process of color formation is too complex. The addition of ground red pepper can contribute to color development. The L*, a*, and b* values of sausages are influenced by fat content, temperature, and ripening time, as well as by the interaction of temperature and ripening period. A high ripening temperature and low-fat content lead to better color development [84]. The analysis of Pis color lightness (L*) showed a decrease in the value observed in the Spanish fermented sausage “Galician chorizo” and Petrovac sausage [85,86]. Škaljac et al. [86] explained the decrease in color lightness value (L*) at the beginning of the process with the influence of the smoking process itself, which was not the case in our study. Bozkut and Bairam [87] also state that the decrease in the L* value is associated with the formation of a dark sausage color. Pérez-Alvarez et al. [88] concluded that moisture loss affects the decrease in the L* value, which was previously found during the ripening process [89]. Rosmini et al. [90] proved that the addition of table salt and paprika to a model system of fermented dried sausages affected the decrease in the L* value, while the addition of water caused the increase. A decrease in the L* value during ripening was observed both when measuring the color of the cross-sectional and the surface color of the sausage without casing. Similarly, the red color (a*) of Pis decreases during the drying process, which is in agreement with earlier reports [85,86]. Partial or complete denaturation of nitrosomyoglobin due to the production of lactic acid, and the reduction in water content in the product at the beginning of the fermentation process was the reason for the decrease in the a* value [87,88]. Fermented dry sausages with a lower NaCl content and lower pH have a higher proportion of red color (a*) on cross-sectional surfaces [91]. This was similarly detected for the higher proportion of yellow coloring. During the drying process of Pirot ironed sausage, the values of the proportion of a yellow color (b*) on the surface and in the cross-section of the sausages generally decreased. Moreover, the obtained results for the decreased proportion of a yellow color (b*) in Pis are due to the consumption of O_2_ by microorganisms, which in turn leads to the reduction in oxymyoglobin and the development of a yellow color [87]. The color transformation of sausages occurs faster at higher temperatures than at lower temperatures, which is most noticeable in the change of the b* value [84]. Papadima and Bloukas [92] confirmed that fat level and storage time significantly affect the L*, a*, and b* values of Greek sausages, while the fat content is proportional to the increase in the L* value.

The texture of fermented sausages is a sensory characteristic that develops during the ripening due to processes that lead to the formation of a firmer consistency, as well as due to the process of proteolysis, which leads to the softening and better chewability of the product. During ripening, the compactness of the filling is formed, i.e., the firm consistency of the sausages. The firmness and toughness of sausages depend on protein coagulation at low pH, but also on reduced moisture content [87]. Fermented sausages with a lower percentage of fatty tissue are firmer [93]. In our study, firmness and toughness increased during ripening, which was comparable to the results for Felino salami [94]. In the study of the texture of dry Italian salami during 55 days of drying and ripening, a significantly lower breaking strength was found compared to our study [95]. Additionally, in the research of Ikonić et al. [72], a lower breaking force was determined during the ripening of the Petrovac sausage. It was observed that the increase in measured texture values was slower in sausages with a longer maturation (Italian sausages), but faster in sausages with a shorter maturation (Spanish chorizo sausages) [96,97]. During the ripening of goat sausages, an increase in firmness was observed in all samples with different proportions of goat meat [80]. Madruga and Bressan [98] proved that the meat of old and emaciated goats can be used in finely chopped cured sausages as the only source of meat. Fat reduction in dry-fermented sausages significantly affected the texture of the sausage. A significant increase in hardness due to fat reduction was observed only at longer ripening times due to the loss of moisture content. In addition, fat reduction was responsible for the increase in toughness regardless of the ripening time [99].

## 5. Conclusions

The very specific method of production makes “Pirot ‘iron’ sausage“ a unique product, and learning about its microbiome for the first time would improve our knowledge of it for other producers on the market and for science in general. The synergy of data obtained by using both traditional and NGS methods allows for the most accurate bacterial identification. Moreover, to our knowledge, this is the first report based on integrative methods that include bacterial community shifts in combination with the chemical–physical parameters of this traditional Serbian brand. It is interesting to note that the succession of the bacterial community achieved, along with a *Lactobacillus* increase (*Latilactobacillus* and *Dellaglioa* according to the new reclassification, with the most represented species such as *L. sakei*, *D. algidus*, and *L. graminis*), a simultaneous decrease in the genera *Photobacterium* and *Weissella*. Although a gradual decline of *Weissella* was observed during the ripening, generally *Lactobacillus* (*Latilactobacillus, Lactiplantibacillus* and, *Dellaglioa*) and *Weissella* can be considered the most important LAB. Although an evaluation of the number of detected sequences for *Photobacterium* suggests the presence of *P. carnosum*, *P. phosphoreum*, *P. iliopiscarium*, and *P. piscicola*, more precise techniques will need to be used in the future, including long read sequencing. However, *Photobacterium* is already known to be a ubiquitous psychrophilic bacterium and its effects on meat spoilage remain to be elucidated in further studies.

## Figures and Tables

**Table 1:** Alpha diversity within the analyzed intestine and sausage samples during different time points of ripening, presented at the phylum, family, genus, and OTU level.

Sample	Intestine/Day of Ripening	OBS	Chao1	SE Chao1	ACE	SE ACE	Shannon	Simpson	Inv Simpson	Fisher	Taxa Hierarchy
Z1	Small bovine intestine	279	279.5	0.99	280.0	7.75	3.8	1.0	24.0	33.9	OTU
		95	95.0	0.00	95.0	4.64	2.8	0.9	10.9	10.1	Genus
		42	42.0	0.00	42.0	2.76	2.0	0.8	5.6	4.1	Family
		8	8.0	0.00	8.0	1.22	0.8	0.5	2.0	0.7	Phylum
Z2	0	126	126.3	0.66	127.1	5.28	2.7	0.9	6.9	14.3	OTU
Z3	2nd	132	133.7	2.20	133.5	5.52	2.8	0.9	8.5	14.7	
Z4	7th	118	121.0	3.18	120.8	5.37	2.7	0.9	8.4	13.5	
Z5	10th	132	139.0	6.65	136.6	5.06	2.8	0.9	8.5	14.5	
Z6	14th	121	121.8	1.26	122.7	5.36	2.5	0.9	6.9	13.0	
Z7	21st	127	128.9	2.26	130.0	5.07	2.6	0.9	7.6	14.2	
											
Z2	0	38	38.0	0.00	38.0	3.01	1.4	0.6	2.4	3.7	Genus
Z3	2nd	47	47.3	0.74	47.6	3.38	1.5	0.7	3.0	4.6	
Z4	7th	40	40.0	0.16	40.3	3.15	1.5	0.7	3.3	4.0	
Z5	10th	38	38.0	0.16	38.5	2.50	1.5	0.7	3.2	3.6	
Z6	14th	40	40.1	0.44	41.1	3.15	1.4	0.7	3.2	3.8	
Z7	21st	40	40.2	0.62	41.1	3.00	1.5	0.7	3.2	3.9	
											
Z2	0	26	26.0	0.00	26.0	2.43	1.3	0.6	2.4	2.5	Family
Z3	2nd	31	31.0	0.16	31.3	2.62	1.4	0.6	2.8	2.9	
Z4	7th	28	28.0	0.16	28.3	2.50	1.4	0.7	3.1	2.7	
Z5	10th	23	23.0	0.00	23.0	1.98	1.4	0.7	3.1	2.1	
Z6	14th	27	27.1	0.49	28.6	2.61	1.3	0.7	3.1	2.5	
Z7	21st	25	25.3	0.73	26.5	2.51	1.4	0.7	3.1	2.3	
											
Z2	0	7	7.0	0.00	7.0	0.93	0.9	0.5	2.1	0.6	Phylum
Z3	2nd	7	7.0	0.00	7.0	1.20	0.9	0.6	2.3	0.6	
Z4	7th	7	7.0	0.00	7.0	1.31	1.0	0.6	2.4	0.6	
Z5	10th	7	7.0	0.00	7.0	0.93	0.8	0.5	2.2	0.6	
Z6	14th	8	8.0	0.00	8.0	1.22	0.9	0.6	2.2	0.6	
Z7	21st	6	6.0	0.00	6.0	0.91	0.7	0.5	1.9	0.5	

**Table 2:** Common and unique species diversity within the analyzed intestine and sausage samples during different time points of ripening by culturable approach.

Isolates Origin	No. of Total Isolates/Unique Species	Total Number of Unique Species for Sample(s)
Z1 (intestine)	8	5
Z2 (0 day)	28	8
Z3 (2nd day)	26	9
Z4 (7th day)	28	10
Z5 (10th day)	24	8
Z6 (14th day)	20	7
Z7 (21st day)	23	6
**∑**	**157**	**32**
Z1 Z2 Z3 Z4 Z5 Z6 Z7	1	*Latilactobacillus sakei* (basonym *Lactobacillus sakei*)
Z2 Z3 Z4 Z5 Z6 Z7	1	*Leuconostoc mesenteroides*
Z1 Z2 Z5 Z6	1	*Enterococcus faecium*
Z1 Z2 Z7	1	*Bacillus toyonensis*/*wiedmannii*/*sanguinis*
Z3 Z5 Z6	1	*Carnobacterium divergens*
Z2 Z7	1	*Bacillus tropicus*/*paramycoides*/*luti*
Z3 Z4	1	*Macrococcus canis*
Z6 Z7	1	*Lactococcus garvieae*
Z1	2	*Enterococcus hirae*; *Enterococcus durans*
Z2	3	*Corynebacterium lipophiloflavum*; *Marichromatium purpuratum*; *Macrococcus caseolyticus*
Z3	5	*Pectobacterium wasabiae*; *Hafnia alvei*; *Serratia proteamaculans*; *Bacillus licheniformis*; *Hafnia paralvei*
Z4	7	*Kocuria kristinae*; *Pantoea agglomerans*/*Enterobacter ludwigii*/*Enterobacter cloacae* subsp. *dissolvens*; *Enterococcus pallens*; *Weissella cibaria*; *Citrobacter murliniae*; *Lactococcus lactis* subsp. *hordniae*; *Enterobacter aerogenes*
Z5	4	*Carnobacterium maltaromaticum*; *Moraxella osloensis*; *Latilactobacillus sakei* subsp. *carnosus* (basonym *Lactobacillus sakei* subsp. *carnosus*); *Enterococcus casseliflavus*
Z6	2	*Shigella sonnei*; *Leuconostoc pseudomesenteroides*
Z7	1	*Leuconostoc rapi*

**Table 3:** The colorimetric and textural characterization throughout the ripening of “Pirot ‘ironed’ sausage.

Ripening	The Color Parameters		
	L*	a*	b*
	The Surface Color of the Sausage with Casing		
0 day	33.3 ± 1.71 ^a^	14.9 ± 0.32 ^a^	4.4 ± 0.20 ^a^
7th day	29.6 ± 1.57 ^ab^	12.2 ± 0.50 ^b^	2.8 ± 0.41 ^ab^
14th day	21.4 ± 0.94 ^c^	9.8 ± 0.82 ^c^	1.8 ± 0.26 ^bc^
21st day	22.8 ± 1.81 ^bc^	8.4 ± 0.50 ^cd^	−0.7 ± 1.02 ^cd^
28th day	25.0 ± 4.95 ^bc^	7.0 ± 0.80 ^d^	−0.04 ± 1.55 ^d^
	**The surface color of the sausage without casing**		
0 day	-	-	-
7th day	-	-	-
14th day	24.3 ± 0.83 ^a^	10.2 ± 0.50 ^a^	1.7 ± 0.97 ^a^
21st day	23.2 ± 1.04 ^ab^	8.8 ± 0.57 ^ab^	−0.4 ± 0.07 ^b^
28th day	21.4 ± 0.94 ^b^	7.7 ± 1.46 ^b^	−0.71 ± 0.36 ^b^
	**The cross-sectional surface color of the sausage**		
	L*	a*	b*
0 day	25.1 ± 1.8 ^ab^	25.4 ± 0.47 ^a^	15.9 ± 0.44 ^a^
7th day	25.3 ± 0.60 ^a^	25.7 ± 0.92 ^a^	15.5 ± 1.29 ^a^
14th day	24.2 ± 0.46 ^ab^	19.3 ± 0.53 ^b^	6.0 ± 0.98 ^b^
21st day	23.9 ± 1.34 ^ab^	16.3 ± 0.67 ^c^	1.8 ± 0.37 ^c^
28th day	22.1 ± 1.15 ^b^	12.9 ± 1.77 ^d^	0.5 ± 1.13 ^c^
	**Textural parameters**		
	Firmness (N)	Toughness (N s)	
0 day	-	-	
7th day	-	-	
14th day	20.3 ± 4.12 ^c^	86.4 ± 14.27 ^c^	
21st day	31.8 ± 1.98 ^b^	165.7 ± 5.31 ^b^	
28th day	42.9 ± 4.98 ^a^	231.0 ± 20.17 ^a^	

Values within the same column during ripening followed by different letters are significantly different (*p* < 0.05), according to Tukey’s HSD test. L*—lightness; a*—redness; b*—yellowness.

## Data Availability

All data have been deposited in the NCBI database as BioProject ID: PRJNA910955 (under the accessions from SAMN32154226 to SAMN32154232). https://www.ncbi.nlm.nih.gov/sra?linkname=bioproject_sra_all&from_uid=910955 (accessed on 21 December 2022).

## Supplementary Materials

The following supporting information can be downloaded at: https://www.mdpi.com/article/10.3390/foods12030664/s1, Figure S1: The heatmap created with Bray–Curtis dissimilarities at the OTU level showing the variability within the samples; Figure S2: Venn diagram of common species between samples (Z2–Z6) throughout the ripening of “Pirot ‘ironed’ sausage”; Table S1: A total number of the reads retention; Table S2: Homology of the identified OTUs annotated based on the BLAST*n* best bit score in the NCBI 16S database; Table S3: The list of total samples of culturable bacterial communities; Table S4: Physical–chemical and technological parameters as the mean differences of statistical significance throughout the ripening of “Pirot ‘ironed’ sausage”.
