# Supplementary material for: Bacteriobiota and Chemical Changes during the Ripening of Traditional Fermented “Pirot ‘Ironed’ Sausage”
Source: Foods. 2023 Feb 3;12(3):664. doi: 10.3390/foods12030664 (PMC9913956; doi:10.3390/foods12030664)
Supplement: Supplementary file 1 [file foods-12-00664-s001.zip › foods-2146666-supplementary.pdf]

Article

Bacteriobiota and Chemical Changes during the Ripening of Traditional Fermented "Pirot 'Ironed' Sausage"

Svetlana Bogdanović [1], Slaviša Stanković [2], Tanja Berić [2], Igor Tomasevic [3,4,*], Volker Heinz [4], Nino Terjung [4] and Ivica Dimkić [2,*]

[1] Agriculture and Food College of Applied Studies, Ćirila i Metodija 1, 18400 Prokuplje, Serbia
[2] Faculty of Biology, University of Belgrade, Studentski trg 16, 11158 Belgrade, Serbia
[3] Faculty of Agriculture, University of Belgrade, Nemanjina 6, 11080 Belgrade, Serbia
[4] DIL German Institute of Food Technologies, Prof.-v.-Klitzing-Str. 7, 49610 Quakenbrueck, Germany
* Correspondence: tbigor@agrif.bg.ac.rs (I.T.); ivicad@bio.bg.ac.rs (I.D.)

Citation: Bogdanović, S.; Stanković, S.; Berić, T.; Tomasevic, I.; Heinz, V.; Terjung, N.; Dimkić, I. Bacteriobiota and Chemical Changes during the Ripening of Traditional Fermented "Pirot 'Ironed' Sausage". *Foods* **2023**, *12*, x. https://doi.org/10.3390/xxxxx

Academic Editors: Jasna Mrvčić and Damir Stanzer

Received: 22 December 2022
Revised: 23 January 2023
Accepted: 29 January 2023
Published: 3 February 2023

Copyright: © 2023 by the authors. Submitted for possible open access publication under the terms and conditions of the Creative Commons Attribution (CC BY) license (https://creativecommons.org/licenses/by/4.0/).

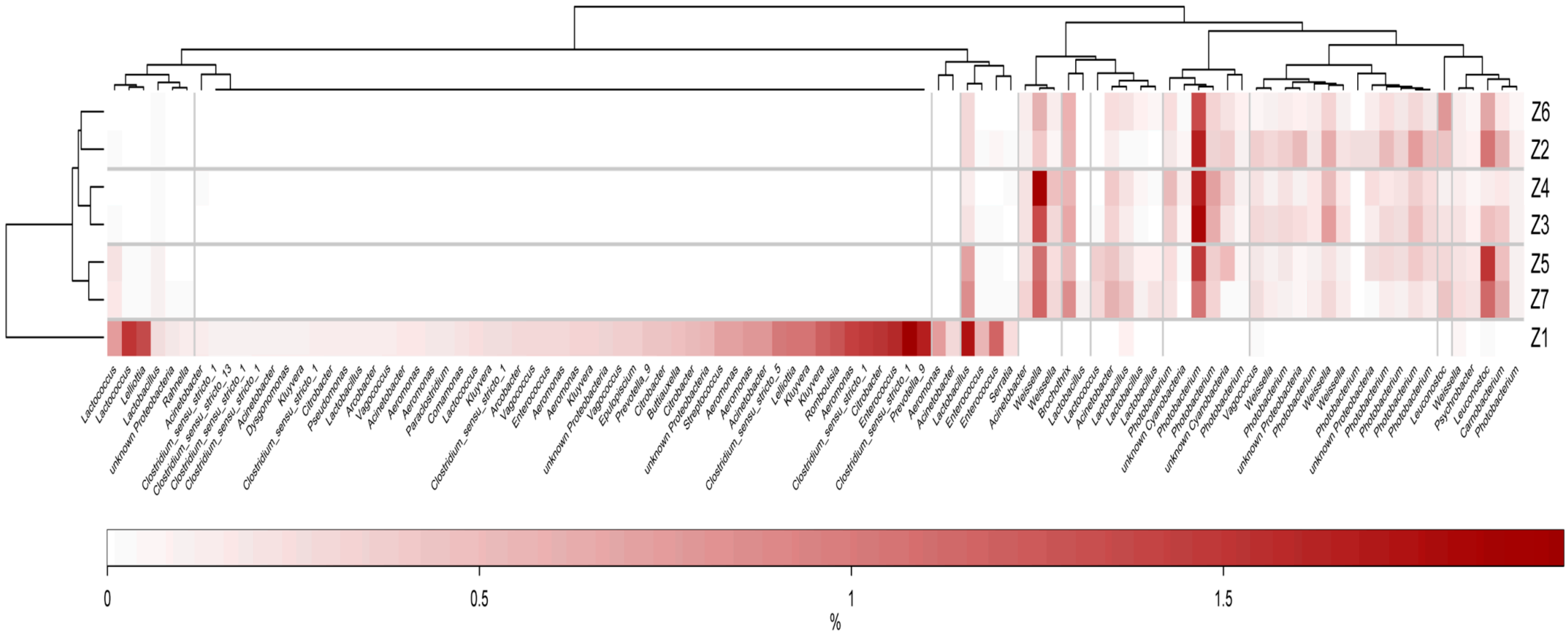

Figure S1. The heatmap created with Bray-Curtis dissimilarities at the OTU level showing the variability within the samples.

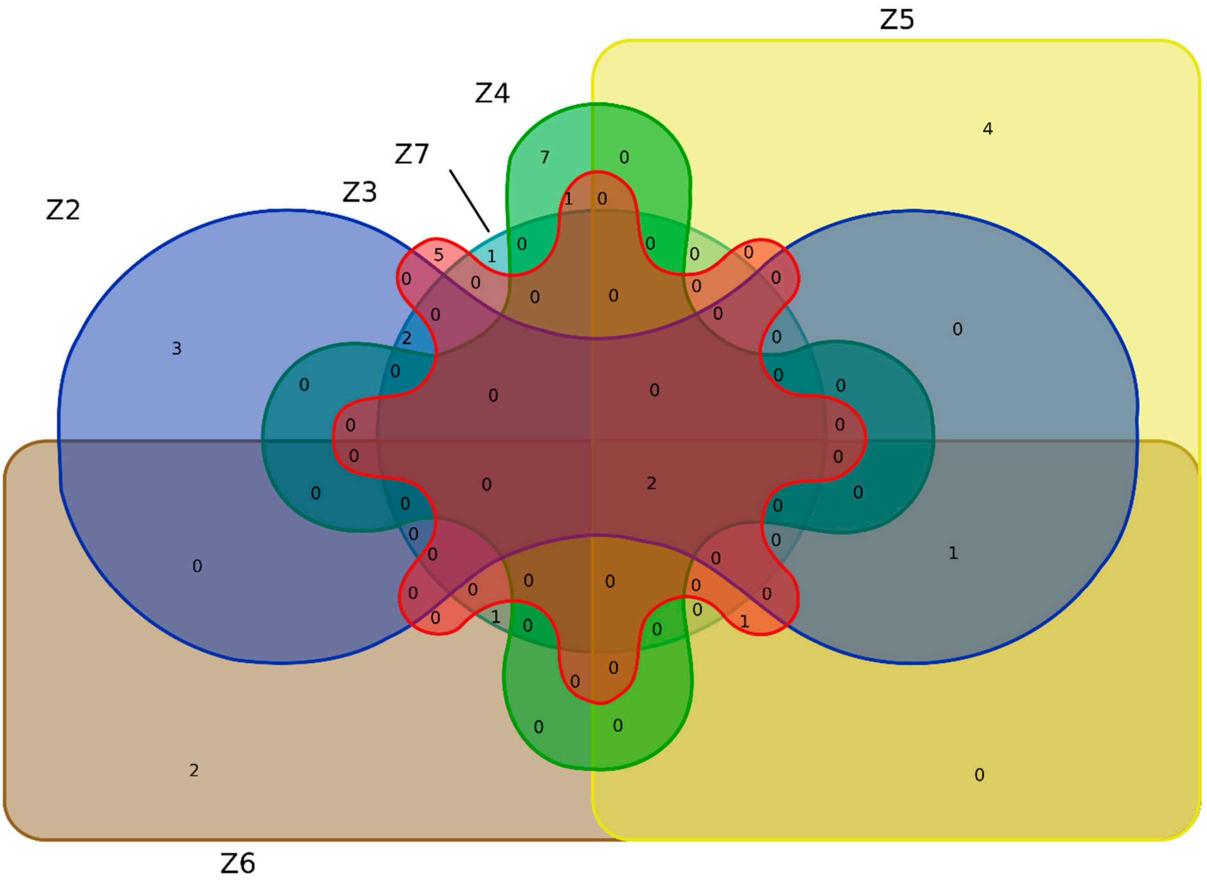

Figure S2. Venn diagram of common species between samples (Z2-Z6) throughout the ripening of "Pirot 'ironed' sausage".

Table S1. A total number of the reads retention.

Sample	Input	Primer remove	Quality filter	Denoised F	Denoised R	Merged	Chimera remove	Final % of reads
Z1	169672	168669	156105	154649	154959	148160	127677	75.25
Z2	127184	117614	109826	109178	109328	105343	96909	76.20
Z3	148238	142399	133327	132649	132808	127148	114558	77.28
Z4	107113	104776	99454	98977	99092	95421	86161	80.44
Z5	166600	162465	152607	151885	152033	145402	128808	77.32
Z6	189000	184973	174132	173236	173503	166585	146679	77.61
Z7	145762	141171	133262	132584	132710	126288	108690	74.57
Total/average	1053569	1022067	958713	953158	954433	914347	809482	76.95

Table S2. Homology of the identified OTUs annotated based on the BLAST*n* best bit score in the NCBI 16S database.

No.	Qseq ID	Sseq ID	Ident. (%)	Length	E value	Bit score	Species name
1	seq_001	gi|1397641705|ref|NR_156814.1|	100	427	0	789	*Photobacterium carnosum*
16	seq_004	gi|1397641705|ref|NR_156814.1|	99.766	427	0	784	
161	seq_033	gi|1397641705|ref|NR_156814.1|	99.766	427	0	784	
196	seq_040	gi|1397641705|ref|NR_156814.1|	99.532	427	0	778	
216	seq_044	gi|1397641705|ref|NR_156814.1|	99.766	427	0	784	
231	seq_047	gi|1397641705|ref|NR_156814.1|	99.532	427	0	778	
256	seq_052	gi|1397641705|ref|NR_156814.1|	99.532	427	0	778	
336	seq_068	gi|1397641705|ref|NR_156814.1|	99.766	427	0	784	
376	seq_076	gi|1397641705|ref|NR_156814.1|	99.532	427	0	778	
526	seq_106	gi|1397641705|ref|NR_156814.1|	99.766	427	0	784	
531	seq_107	gi|1397641705|ref|NR_156814.1|	99.766	427	0	784	
1176	seq_236	gi|1397641705|ref|NR_156814.1|	100	418	0	773	
1291	seq_259	gi|1397641705|ref|NR_156814.1|	100	427	0	789	
41	seq_009	gi|631252986|ref|NR_114184.1|	100	427	0	789	*Photobacterium phosphoreum*
276	seq_056	gi|631252986|ref|NR_114184.1|	99.766	427	0	784	
56	seq_012	gi|631252986|ref|NR_114184.1|	99.766	427	0	784	
46	seq_010	gi|631250795|ref|NR_111990.1|	100	427	0	789	*Photobacterium iliopiscarium*
51	seq_011	gi|631250795|ref|NR_111990.1|	99.766	427	0	784	
151	seq_031	gi|631250795|ref|NR_111990.1|	99.532	427	0	778	
406	seq_082	gi|631250795|ref|NR_111990.1|	99.766	427	0	784	
71	seq_015	gi|672239090|ref|NR_125679.1|	100	427	0	789	*Photobacterium piscicola*
11	seq_003	gi|631252623|ref|NR_113821.1|	100	427	0	789	*Lactobacillus sakei*
441	seq_089	gi|631252623|ref|NR_113821.1|	99.766	427	0	784	
446	seq_090	gi|631252623|ref|NR_113821.1|	99.766	427	0	784	
511	seq_103	gi|631252623|ref|NR_113821.1|	99.766	427	0	784	
716	seq_144	gi|631252623|ref|NR_113821.1|	99.766	427	0	784	
1876	seq_376	gi|631252623|ref|NR_113821.1|	100	427	0	789	
6	seq_002	gi|265678315|ref|NR_028617.1|	100	427	0	789	*Lactobacillus algidus*
1496	seq_300	gi|265678315|ref|NR_028617.1|	96.487	427	0	706	
1766	seq_354	gi|265678315|ref|NR_028617.1|	100	427	0	789	
1661	seq_333	gi|265678315|ref|NR_028617.1|	100	420	0	776	
121	seq_025	gi|343201712|ref|NR_042438.1|	99.766	427	0	784	*Lactobacillus graminis*
366	seq_074	gi|343201712|ref|NR_042438.1|	99.532	427	0	778	
881	seq_177	gi|343201712|ref|NR_042438.1|	99.532	427	0	778	
246	seq_050	gi|559795383|ref|NR_104976.1|	100	427	0	789	*Lactobacillus fuchuensis*
616	seq_124	gi|672238974|ref|NR_125563.1|	100	427	0	789	*Lactobacillus nenjiangensis*
1151	seq_231	gi|645319700|ref|NR_117065.1|	100	427	0	789	*Lactobacillus ultunensis*
1506	seq_302	gi|1491505234|ref|NR_159082.1|	99.766	427	0	784	*Lactobacillus allii*

1676	seq_336	gi|631253167|ref|NR_114365.1|	99.063	427	0	767	*Lactobacillus iwatensis*
1761	seq_353	gi|343198639|ref|NR_043148.1|	100	427	0	789	*Lactobacillus oligofermentans*
1816	seq_364	gi|631253053|ref|NR_114251.1|	98.829	427	0	761	*Lactobacillus senmaizukei*
2006	seq_402	gi|1199303358|ref|NR_147709.1|	98.829	427	0	761	*Lactobacillus wasatchensis*
21	seq_005	gi|959494902|ref|NR_133769.1|	100	427	0	789	
2001	seq_401	gi|959494902|ref|NR_133769.1|	99.766	427	0	784	*Leuconostoc gelidum*
1591	seq_319	gi|959494902|ref|NR_133769.1|	98.656	372	0	660	
61	seq_013	gi|1269801505|ref|NR_074997.2|	100	427	0	789	
101	seq_021	gi|1441204190|ref|NR_157602.1|	100	427	0	789	*Leuconostoc mesenteroides*
791	seq_159	gi|631250774|ref|NR_109004.1|	99.766	427	0	784	*Leuconostoc pseudomesenteroides*
166	seq_034	gi|343200124|ref|NR_040811.1|	100	427	0	789	*Leuconostoc carnosum*
1011	seq_203	gi|343202334|ref|NR_042620.1|	100	427	0	789	*Leuconostoc holzapfelii*
1786	seq_358	gi|631252057|ref|NR_113255.1|	100	427	0	789	*Leuconostoc lactis*
26	seq_006	gi|566085118|ref|NR_108858.1|	99.061	426	0	765	
146	seq_030	gi|566085118|ref|NR_108858.1|	98.826	426	0	760	
316	seq_064	gi|566085118|ref|NR_108858.1|	98.826	426	0	760	*Weissella fabalis*
321	seq_065	gi|566085118|ref|NR_108858.1|	98.826	426	0	760	
1886	seq_378	gi|566085118|ref|NR_108858.1|	99.061	426	0	765	
96	seq_020	gi|265678736|ref|NR_029041.1|	100	427	0	789	
236	seq_048	gi|265678736|ref|NR_029041.1|	99.766	427	0	784	*Weissella koreensis*
296	seq_060	gi|265678736|ref|NR_029041.1|	99.766	427	0	784	
832	seq_167	gi|310975060|ref|NR_036924.1|	100	427	0	789	*Weissella cibaria*
191	seq_039	gi|631252761|ref|NR_113959.1|	100	427	0	789	
516	seq_104	gi|631252761|ref|NR_113959.1|	99.766	427	0	784	*Lactococcus raffinolactis*
901	seq_181	gi|631252761|ref|NR_113959.1|	99.766	427	0	784	
273	seq_055	gi|631252762|ref|NR_113960.1|	100	427	0	789	*Lactococcus lactis*
358	seq_072	gi|631252070|ref|NR_113268.1|	100	427	0	789	*Lactococcus garvieae*
76	seq_016	gi|343204087|ref|NR_043739.1|	100	427	0	789	*Lactococcus piscium*
156	seq_032	gi|631252600|ref|NR_113798.1|	100	427	0	789	*Carnobacterium divergens*
696	seq_140	gi|343201367|ref|NR_042093.1|	99.532	427	0	780	*Carnobacterium gallinarum*
991	seq_199	gi|636560400|ref|NR_116460.1|	100	427	0	789	*Carnobacterium jeotgali*
126	seq_026	gi|631252389|ref|NR_113587.1|	100	427	0	789	*Brochothrix thermosphacta*
176	seq_036	gi|631252148|ref|NR_113346.1|	98.135	429	0	747	
291	seq_059	gi|631252148|ref|NR_113346.1|	97.902	429	0	741	*Acinetobacter lwoffii*
1346	seq_270	gi|631252148|ref|NR_113346.1|	100	428	0	791	
326	seq_066	gi|645320413|ref|NR_117624.1|	99.065	428	0	769	
456	seq_092	gi|645320413|ref|NR_117624.1|	99.533	428	0	780	*Acinetobacter johnsonii*
566	seq_114	gi|645320413|ref|NR_117624.1|	99.766	428	0	785	

586	seq_118	gi|645320413|ref|NR_117624.1|	99.766	428	0	785	
916	seq_184	gi|645320413|ref|NR_117624.1|	99.533	428	0	780	
921	seq_185	gi|645320413|ref|NR_117624.1|	99.065	428	0	769	
986	seq_198	gi|645320413|ref|NR_117624.1|	99.766	428	0	785	
1051	seq_211	gi|645320413|ref|NR_117624.1|	99.533	428	0	780	
1071	seq_215	gi|645320413|ref|NR_117624.1|	98.832	428	0	763	
751	seq_151	gi|1341394069|ref|NR_153741.1|	99.533	428	0	780	*Acinetobacter celticus*
1701	seq_341	gi|1341394069|ref|NR_153741.1|	98.832	428	0	763	
846	seq_170	gi|645320481|ref|NR_117677.1|	97.436	429	0	730	*Acinetobacter baumannii*
1741	seq_349	gi|645320481|ref|NR_117677.1|	97.669	429	0	736	
651	seq_131	gi|961554986|ref|NR_133953.1|	97.902	429	0	741	*Acinetobacter gandensis*
1386	seq_278	gi|961554986|ref|NR_133953.1|	100	428	0	791	
1566	seq_314	gi|961554986|ref|NR_133953.1|	98.14	429	0	747	
976	seq_196	gi|1137647852|ref|NR_145641.1|	99.533	428	0	780	*Acinetobacter albensis*
1276	seq_256	gi|1137647852|ref|NR_145641.1|	98.598	428	0	758	
806	seq_162	gi|1146059109|ref|NR_145841.1|	99.766	428	0	785	*Acinetobacter movanagherensis*
891	seq_179	gi|1146059109|ref|NR_145841.1|	98.364	428	0	752	
941	seq_189	gi|645320415|ref|NR_117626.1|	100	428	0	791	*Acinetobacter guillouiae*
1261	seq_253	gi|645320419|ref|NR_117629.1|	99.533	428	0	780	*Acinetobacter tjernbergiae*
1296	seq_260	gi|1230874683|ref|NR_148844.1|	97.196	428	0	725	*Acinetobacter dispersus*
1356	seq_272	gi|1397641879|ref|NR_156989.1|	98.832	428	0	763	*Acinetobacter defluvii*
691	seq_139	gi|1277396356|ref|NR_152069.1|	98.598	428	0	758	*Acinetobacter pragensis*

Table S3. The list of total samples of culturable bacterial communities.

Coded name	Species name	Closest accession number
	**Intestine**	
C1	*Enterococcus faecium*	NR 115764
C2	*Enterococcus faecium*	NR 115764
C3	*Enterococcus durans*	NR 113900
C4	*Enterococcus durans*	NR 113900
C5	*Lactobacillus sakei*	NR 113821
haC1	*Enterococcus hirae*	NR 114783
haC3	*Enterococcus faecium*	NR 115764
haC8	*Bacillus toyonensis/wiedmannii/sanguinis*	NR 121761/NR 152692/NR 175555
	**0 day**	
0/1	*Leuconostoc mesenteroides*	NR 074957
0/2	*Leuconostoc mesenteroides*	NR 074957
0/5	*Leuconostoc mesenteroides*	NR 074957
0/6	*Leuconostoc mesenteroides*	NR 074957
0/7	*Leuconostoc mesenteroides*	NR 074957
0/8	*Leuconostoc mesenteroides*	NR 074957
0/9	*Leuconostoc mesenteroides*	NR 074957
0/10	*Leuconostoc mesenteroides*	NR 074957
0/11	*Leuconostoc mesenteroides*	NR 074957
0/12	*Leuconostoc mesenteroides*	NR 074957
0/13	*Leuconostoc mesenteroides*	NR 074957
0/14	*Leuconostoc mesenteroides*	NR 074957
0/15	*Leuconostoc mesenteroides*	NR 074957
0/16	*Leuconostoc mesenteroides*	NR 074957
0/17	*Leuconostoc mesenteroides*	NR 074957
0/18	*Leuconostoc mesenteroides*	NR 074957
0/19	*Leuconostoc mesenteroides*	NR 074957
0/20	*Leuconostoc mesenteroides*	NR 074957
0/21	*Leuconostoc mesenteroides*	NR 074957
0/22	*Leuconostoc mesenteroides*	NR 074957
ha0/0	*Lactobacillus sakei*	NR 113821
ha0/1	*Bacillus tropicus/paramycoides/luti*	NR 157736/NR 157734/NR 157730
ha0/2	*Marichromatium purpuratum*	NR 116469
ha0/3	*Bacillus toyonensis/wiedmannii/sanguinis*	NR 121761/NR 152692/NR 175555
ha0/10	*Enterococcus faecium*	NR 115764
ha0/12	*Bacillus tropicus/paramycoides/luti*	NR 157736/NR 157734/NR 157730
ha0/13	*Macrococcus caseolyticus*	NR 119262
ha0/20	*Corynebacterium lipophiloflavum*	NR 026370
	**2nd day**	
2/1	*Leuconostoc mesenteroides*	NR 074957
2/2	*Leuconostoc mesenteroides*	NR 074957

2/3	*Leuconostoc mesenteroides*	NR 074957
2/5	*Leuconostoc mesenteroides*	NR 074957
2/7	*Leuconostoc mesenteroides*	NR 074957
2/8	*Leuconostoc mesenteroides*	NR 074957
2/9	*Leuconostoc mesenteroides*	NR 074957
2/10	*Leuconostoc mesenteroides*	NR 074957
2/11	*Leuconostoc mesenteroides*	NR 074957
2/12	*Leuconostoc mesenteroides*	NR 074957
2/13	*Leuconostoc mesenteroides*	NR 074957
2/15	*Leuconostoc mesenteroides*	NR 074957
2/16	*Leuconostoc mesenteroides*	NR 074957
2/17	*Leuconostoc mesenteroides*	NR 074957
2/18	*Leuconostoc mesenteroides*	NR 074957
2/19	*Leuconostoc mesenteroides*	NR 074957
2/20	*Leuconostoc mesenteroides*	NR 074957
ha2/1	*Lactobacillus sakei*	NR 113821
ha2/2	*Serratia proteamaculans*	NR 025341
ha2/4	*Hafnia alvei*	NR 044729
ha2/7	*Hafnia paralvei*	NR 116898
ha2/9	*Carnobacterium divergens*	NR 113798
ha2/10	*Serratia proteamaculans*	NR 025341
ha2/13	*Bacillus licheniformis*	NR 118996
ha2/17	*Macrococcus canis*	NR 156154
ha2/20	*Pectobacterium wasabiae*	NR 118294
	**7th day**	
7/1	*Leuconostoc mesenteroides*	NR 074957
7/2	*Leuconostoc mesenteroides*	NR 074957
7/3	*Leuconostoc mesenteroides*	NR 074957
7/4	*Leuconostoc mesenteroides*	NR 074957
7/5	*Leuconostoc mesenteroides*	NR 074957
7/6	*Leuconostoc mesenteroides*	NR 074957
7/7	*Leuconostoc mesenteroides*	NR 074957
7/8	*Leuconostoc mesenteroides*	NR 074957
7/9	*Leuconostoc mesenteroides*	NR 074957
7/10	*Leuconostoc mesenteroides*	NR 074957
7/11	*Leuconostoc mesenteroides*	NR 074957
7/12	*Leuconostoc mesenteroides*	NR 074957
7/14	*Weissella cibaria*	NR 036924
7/15	*Leuconostoc mesenteroides*	NR 074957
7/16	*Leuconostoc mesenteroides*	NR 074957
7/17	*Leuconostoc mesenteroides*	NR 074957
7/18	*Leuconostoc mesenteroides*	NR 074957
7/19	*Leuconostoc mesenteroides*	NR 074957

7/20	*Leuconostoc mesenteroides*	NR 074957
ha7/0	*Lactobacillus sakei*	NR 113821
ha7/3	*Citrobacter murliniae*	NR 028688
ha7/7	*Pantoea agglomerans/Enterobacter ludwigii/Enterobacter cloacae* subsp. *dissolvens*	NR 111998/NR 042349/NR 118011
ha7/8	*Enterococcus pallens*	NR 043794
ha7/10	*Lactococcus lactis* subsp. *hordniae*	NR 113958
ha7/11	*Enterobacter aerogenes*	NR 113614
ha7/13	*Macrococcus canis*	NR 156154
ha7/15	*Kocuria kristinae*	NR 026199
ha7/20	*Pantoea agglomerans/Enterobacter ludwigii/Enterobacter cloacae* subsp. *dissolvens*	NR 111998/NR 042349/NR 118011
	**10th day**	
10/1	*Leuconostoc mesenteroides*	NR 074957
10/4	*Leuconostoc mesenteroides*	NR 074957
10/6	*Leuconostoc mesenteroides*	NR 074957
10/7	*Leuconostoc mesenteroides*	NR 074957
10/8	*Leuconostoc mesenteroides*	NR 074957
10/9	*Leuconostoc mesenteroides*	NR 074957
10/10	*Leuconostoc mesenteroides*	NR 074957
10/11	*Leuconostoc mesenteroides*	NR 074957
10/12	*Leuconostoc mesenteroides*	NR 074957
10/13	*Leuconostoc mesenteroides*	NR 074957
10/14	*Leuconostoc mesenteroides*	NR 074957
10/15	*Leuconostoc mesenteroides*	NR 074957
10/16	*Leuconostoc mesenteroides*	NR 074957
10/17	*Leuconostoc mesenteroides*	NR 074957
10/18	*Leuconostoc mesenteroides*	NR 074957
ha10/1	*Enterococcus faecium*	NR 115764
ha10/3	*Lactobacillus sakei*	NR 113821
ha10/4	*Leuconostoc mesenteroides*	NR 074957
ha10/6	*Enterococcus faecium*	NR 115764
ha10/7	*Carnobacterium divergens*	NR 113798
ha10/8	*Enterococcus casseliflavus*	NR 104560
ha10/11	*Moraxella osloensis*	NR 113392
ha10/13	*Carnobacterium maltaromaticum*	NR 044710
ha10/15	*Lactobacillus sakei* subsp. *carnosus*	NR 104208
	**14th day**	
14/3	*Leuconostoc mesenteroides*	NR 074957
14/4	*Leuconostoc pseudomesenteroides*	NR 040814
14/5	*Leuconostoc mesenteroides*	NR 074957
14/6	*Leuconostoc mesenteroides*	NR 074957
14/7	*Leuconostoc mesenteroides*	NR 074957
14/9	*Leuconostoc mesenteroides*	NR 074957
14/10	*Leuconostoc mesenteroides*	NR 074957

14/11	*Leuconostoc mesenteroides*	NR 074957
14/12	*Leuconostoc mesenteroides*	NR 074957
14/13	*Leuconostoc mesenteroides*	NR 074957
14/14	*Leuconostoc mesenteroides*	NR 074957
14/16	*Leuconostoc mesenteroides*	NR 074957
14/17	*Leuconostoc mesenteroides*	NR 074957
14/18	*Leuconostoc mesenteroides*	NR 074957
ha14/1	*Lactobacillus sakei*	NR 113821
ha14/2	*Lactobacillus sakei*	NR 113821
ha14/3	*Carnobacterium divergens*	NR 113798
ha14/4	*Shigella sonney*	NR 104826
ha14/7	*Enterococcus faecium*	NR 115764
ha14/10	*Lactococcus garvieae*	NR 113268
	**21st day**	
21/1	*Leuconostoc mesenteroides*	NR 074957
21/2	*Leuconostoc mesenteroides*	NR 074957
21/3	*Leuconostoc mesenteroides*	NR 074957
21/4	*Leuconostoc mesenteroides*	NR 074957
21/5	*Leuconostoc mesenteroides*	NR 074957
21/6	*Leuconostoc rapi*	NR 136799
21/7	*Leuconostoc mesenteroides*	NR 074957
21/8	*Leuconostoc mesenteroides*	NR 074957
21/10	*Leuconostoc mesenteroides*	NR 074957
21/11	*Leuconostoc mesenteroides*	NR 074957
21/13	*Leuconostoc mesenteroides*	NR 074957
21/14	*Lactococcus garvieae*	NR 113268
21/15	*Leuconostoc mesenteroides*	NR 074957
21/16	*Leuconostoc mesenteroides*	NR 074957
21/17	*Leuconostoc mesenteroides*	NR 074957
ha21/1	*Lactobacillus sakei*	NR 113821
ha21/4	*Bacillus tropicus/paramycoides/luti*	NR 157736/NR 157734/NR 157730
ha21/5	*Lactobacillus sakei*	NR 113821
ha21/6	*Lactobacillus sakei*	NR 113821
ha21/7 (1)	*Bacillus toyonensis/wiedmannii/sanguinis*	NR 121761/NR 152692/NR 175555
ha21/7 (2)	*Bacillus toyonensis/wiedmannii/sanguinis*	NR 121761/NR 152692/NR 175555
ha21/11	*Lactobacillus sakei*	NR 113821
ha21/14	*Lactobacillus sakei*	NR 113821

*the coded names without prefixes represent isolates grown on MRS medium

Table S4. Physical-chemical and technological parameters as the mean differences of statistical significance throughout the ripening of “Pirot ‘ironed’ sausage”.

Dependent Variable	(I) Ripening	(J) Ripening	Mean Difference (I-J)	Sig.
pH	0 day	7th day	0.08111*	0.040
		14th day	0.10333*	0.009
		21st day	0.21333*	0.000
		28th day	0.49333*	0.000
	7th day	14th day	0.02222	0.874
		21st day	0.13222*	0.002
		28th day	0.41222*	0.000
	14th day	21st day	0.11000*	0.006
		28th day	0.39000*	0.000
	21st day	28th day	0.28000*	0.000
a_w	0 day	7th day	0.01144	0.132
		14th day	0.04667*	0.000
		21st day	0.08667*	0.000
		28th day	0.11667*	0.000
	7th day	14th day	0.03522*	0.000
		21st day	0.07522*	0.000
		28th day	0.10522*	0.000
	14th day	21st day	0.04000*	0.000
		28th day	0.07000*	0.000
	21st day	28th day	0.03000*	0.000
Fat (%)	0 day	7th day	-0.27667	0.999
		14th day	-2.67	0.138
		21st day	-2.72667	0.127

The surface color of the sausage with casing

Dependent Variable	(I) Ripening	(J) Ripening	Mean Difference (I-J)	Sig.
L*	0 day	7th day	3.72333	0.451
		14th day	11.91333*	0.002
		21st day	10.58000*	0.004
		28th day	8.33000*	0.019
	7th day	14th day	8.19000*	0.021
		21st day	6.85667	0.056
		28th day	4.60667	0.269
	14th day	21st day	-1.33333	0.968
		28th day	-3.58333	0.486
	21st day	28th day	-2.25	0.825
a*	0 day	7th day	2.76667*	0.002
		14th day	5.08000*	0.000
		21st day	6.49667*	0.000
		28th day	7.87000*	0.000
	7th day	14th day	2.31333*	0.007
		21st day	3.73000*	0.000
		28th day	5.10333*	0.000
	14th day	21st day	1.41667	0.106
		28th day	2.79000*	0.002
	21st day	28th day	1.37333	0.121
b*	0 day	7th day	1.59	0.236
		14th day	2.54000*	0.031
		21st day	5.08667*	0.000

The cross-sectional surface color of the sausage

Dependent Variable	(I) Ripening	(J) Ripening	Mean Difference (I-J)	Sig.
L*	0 day	7th day	-0.23667	0.999
		14th day	0.89667	0.877
		21st day	1.21667	0.714
		28th day	3.02667	0.061
	7th day	14th day	1.13333	0.761
		21st day	1.45333	0.575
		28th day	3.26333*	0.042
	14th day	21st day	0.32	0.997
		28th day	2.13	0.247
	21st day	28th day	1.81	0.381
a*	0 day	7th day	-0.28	0.996
		14th day	6.10000*	0.000
		21st day	9.15333*	0.000
		28th day	12.51667*	0.000
	7th day	14th day	6.38000*	0.000
		21st day	9.43333*	0.000
		28th day	12.79667*	0.000
	14th day	21st day	3.05333*	0.024
		28th day	6.41667*	0.000
	21st day	28th day	3.36333*	0.013
b*	0 day	7th day	0.49667	0.960
		14th day	9.99333*	0.000
		21st day	14.13333*	0.000

		28th day	-4.99333*	0.004
	7th day	14th day	-2.39333	0.206
		21st day	-2.45	0.190
		28th day	-4.71667*	0.006
	14th day	21st day	-0.05667	1.000
		28th day	-2.32333	0.226
	21st day	28th day	-2.26667	0.245
**Moisture (%)**	0 day	7th day	9.12333*	0.000
		14th day	20.52333*	0.000
		21st day	31.53333*	0.000
		28th day	34.39333*	0.000
	7th day	14th day	11.40000*	0.000
		21st day	22.41000*	0.000
		28th day	25.27000*	0.000
	14th day	21st day	11.01000*	0.000
		28th day	13.87000*	0.000
	21st day	28th day	2.86	0.081
**Proteins (%)**	0 day	7th day	-6.11444*	0.000
		14th day	-14.69333*	0.000
		21st day	-23.58333*	0.000
		28th day	-26.67333*	0.000
	7th day	14th day	-8.57889*	0.000
		21st day	-17.46889*	0.000
		28th day	-20.55889*	0.000
	14th day	21st day	-8.89000*	0.000
		28th day	-11.98000*	0.000
	21st day	28th day	-3.09000*	0.026

			28th day	4.42000*	0.001
		7th day	14th day	0.95	0.671
			21st day	3.49667*	0.004
			28th day	2.83000*	0.016
		14th day	21st day	2.54667*	0.030
			28th day	1.88	0.130
		21st day	28th day	-0.66667	0.873
**The surface color of the sausage without casing**	**L***	14th day	21st day	1.12333	0.371
			28th day	2.91667*	0.021
		21st day	28th day	1.79333	0.126
	**a***	14th day	21st day	1.37333	0.258
			28th day	2.54333*	0.039
		21st day	28th day	1.17	0.353
	**b***	14th day	21st day	2.13000*	0.011
			28th day	2.42000*	0.006
		21st day	28th day	0.29	0.829

		28th day	15.42667*	0.000
	7th day	14th day	9.49667*	0.000
		21st day	13.63667*	0.000
		28th day	14.93000*	0.000
	14th day	21st day	4.14000*	0.002
		28th day	5.43333*	0.000
	21st day	28th day	1.29333	0.462
**Firmness (N)**	14th day	21st day	-11.41333*	0.027
		28th day	-22.59000*	0.001
	21st day	28th day	-11.17667*	0.029
**Toughness (N s)**	14th day	21st day	-79.29667*	0.001
		28th day	-144.55667*	0.000
	21st day	28th day	-65.26000*	0.004
